# Risk of cardiovascular and autoimmune disease in people with multiple sclerosis on long-term interferon-β therapy

**DOI:** 10.1093/braincomms/fcaf363

**Published:** 2025-09-22

**Authors:** Bastien Rioux, Feng Zhu, Huah Shin Ng, Yinshan Zhao, Thomas M Caparrotta, William N Whiteley, David P J Hunt, Helen Tremlett

**Affiliations:** Centre for Clinical Brain Sciences, University of Edinburgh, Edinburgh, EH16 4SB, United Kingdom; UK Dementia Research Institute at Edinburgh, University of Edinburgh, Edinburgh, EH16 4SB, United Kingdom; Department of Medicine (Neurology) and the Djavad Mowafaghian Centre for Brain Health, University of British Columbia, Vancouver, British Columbia, V6T 1Z3, Canada; Flinders Health and Medical Research Institute, College of Medicine and Public Health, Flinders University, Adelaide, South Australia, 5042, Australia; SA Pharmacy, SA Health, Government of South Australia, Adelaide, South Australia, 5000, Australia; Department of Medicine (Neurology) and the Djavad Mowafaghian Centre for Brain Health, University of British Columbia, Vancouver, British Columbia, V6T 1Z3, Canada; Centre for Clinical Brain Sciences, University of Edinburgh, Edinburgh, EH16 4SB, United Kingdom; Department of Clinical Pharmacology, NHS Lothian, Edinburgh, EH16 4SB, United Kingdom; Centre for Clinical Brain Sciences, University of Edinburgh, Edinburgh, EH16 4SB, United Kingdom; British Heart Foundation Data Science Centre, Health Data Research UK, London, NW1 2BE, United Kingdom; Centre for Clinical Brain Sciences, University of Edinburgh, Edinburgh, EH16 4SB, United Kingdom; UK Dementia Research Institute at Edinburgh, University of Edinburgh, Edinburgh, EH16 4SB, United Kingdom; Department of Medicine (Neurology) and the Djavad Mowafaghian Centre for Brain Health, University of British Columbia, Vancouver, British Columbia, V6T 1Z3, Canada

**Keywords:** interferon-β, cardiovascular disease, autoimmunity, multiple sclerosis, population data BC

## Abstract

Chronically elevated type I interferons (−β and -α) can induce atherosclerosis and autoimmunity but whether this link translates into adverse events in interferon-β users with multiple sclerosis is unknown. We therefore aimed to determine whether long-term interferon-β exposure increases the risk of cardiovascular and autoimmune disease in a Canadian population-based cohort with linked hospital/physician visits and filled prescriptions. People with multiple sclerosis were included from the most recent of (i) first diagnostic code or disease-modifying therapy or (ii) prescription data availability (1/JAN/1996), and followed until the earliest of outcome, emigration, death or study end (31/DEC/2017). Associations were tested using stratified Cox regressions with time-dependent covariates. The cohort included 19 360 people with multiple sclerosis followed for a median duration of 11.2 years (Q1-Q3: 5.1–18.7), of whom 3138 (16.2%) ever used an interferon-β. Longer interferon-β therapy was associated with a higher incidence of cardiovascular disease (per 5-year longer treatment: hazard ratio = 1.18; 95% confidence interval: 1.02, 1.37; *P* = 0.026) but not with autoimmunity (hazard ratio = 0.74; 95% confidence interval: 0.49, 1.11; *P* = 0.139). This new safety signal should encourage clinicians to optimize cardiovascular prevention in people with multiple sclerosis and may be considered when discussing treatment options in interferon-β users who are at high risk or with established cardiovascular disease.

## Teaser

In this cohort of 19 360 people with multiple sclerosis followed for >20 years, Rioux *et al*. report that longer interferon-β therapy is associated with a higher incidence of cardiovascular disease with a duration-response relationship. They suggest this new safety signal should encourage clinicians to optimize cardiovascular prevention in multiple sclerosis.

## Graphical Abstract

**Graphical Abstract:**
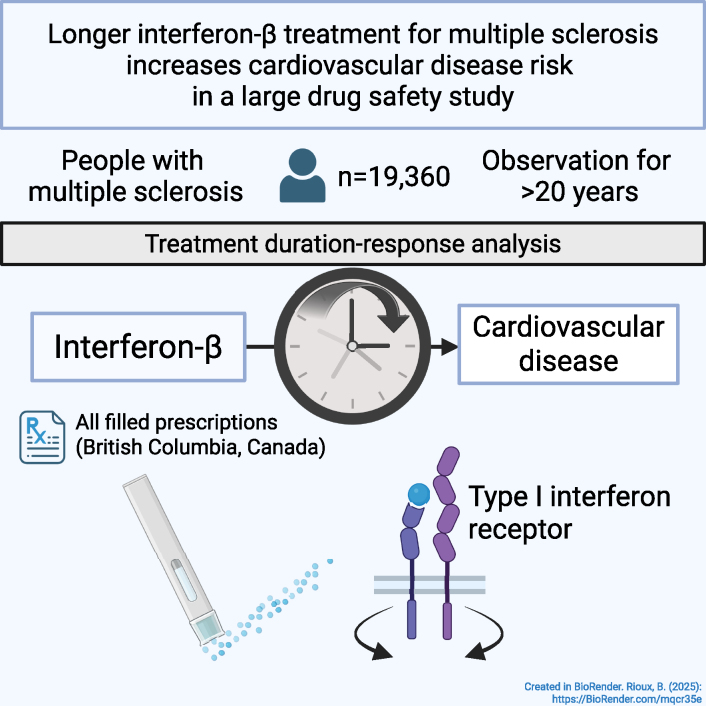

## Introduction

Preclinical evidence suggests that chronically elevated type I interferons (−β and -α) promotes atherosclerosis^1^ and can induce cerebral microangiopathy.^2^ Elevated type I interferons are also increasingly recognized as a hallmark and a major contributor to the onset of autoimmune diseases such as systemic lupus erythematosus,^3^ a condition conferring a higher risk of cardiovascular disease.^4^ As such, there is a biologically plausible association between type I interferons and the risk of cardiovascular and autoimmune disease, but whether this link translates into adverse events in interferon-β users with multiple sclerosis (MS) remains unclear.

Detecting valid cardiovascular and autoimmune safety signals in interferon-β users, however, has previously been challenging. Interferon-β safety data are mostly derived from clinical trials and observational studies with follow-up spanning over months to a few years,^5^ which may be insufficient to detect adverse effects requiring prolonged induction periods such as atherosclerotic disease. Moreover, prior case reports^6-9^ and population-based studies^5,10,11^ reporting on the long-term safety of interferon-β have generated divergent results, partly due to inconsistent control for confounding by indication.^12^ There is, therefore, a pressing need to clarify the long-term safety profile of interferon-β in MS. In this study, we aimed to determine whether long-term exposure to interferon-β increases the risk of cardiovascular and autoimmune disease in a large population-based MS cohort with over two decades of follow-up, using cumulative exposure effects models to control for confounding by indication.

## Methods

This article complies with the RECORD-PE checklist (Supplementary Table 1)^13^ and was approved by the research ethics board at the University of British Columbia (H18–00407/HS21764).

### Data source and study population

We defined a population-based MS cohort in British Columbia (Canada) and extracted diagnoses and dispensed prescriptions from three linked administrative health databases: (i) the Discharge Abstract Database, which records all hospital visits with up to 25 codes per episode (International Classification of Diseases v9/v10-CA), (ii) the Medical Services Plan Payment Information File, which contains billing information submitted by fee-for-service physicians with up to five codes per visit (International Classification of Diseases v9) and (iii) PharmaNet, which captures all filled prescriptions from community/hospital outpatient pharmacies along with unique drug IDs, fill dates and days of supply. In sensitivity analyses, Vital Statistics were also used to define the underlying cause of death (International Classification of Diseases v9/v10).

People with MS, defined from either ≥3 diagnostic codes (v9/v10: 340/G35) or ≥1 disease-modifying therapy (DMT) prescription filled (Supplementary Table 2), were included from the most recent of (i) the first MS/demyelinating diagnostic code (Supplementary Table 3) or DMT prescription filled or (ii) prescription data availability (1/JAN/1996), and followed until the earliest of outcome, emigration, death or study end (31/DEC/2017). The study entry (index date) was shifted forward, if needed, to ensure participants were ≥18 years old and had ≥1 year of residency in the province (to define covariates).

### Definition of exposures, outcomes and covariates

We defined interferon-β therapy intervals as continuous sequences from fill dates plus days of supply followed by a 30-day grace period. Filling a new DMT prescription stopped the previous regimen to avoid combined therapy as per standard practice (Supplementary Fig. 1). We employed the same approach to define exposure to glatiramer acetate, used as negative control exposure.^11^ We tested two primary composite outcomes: (i) cardiovascular disease, composed of ischaemic heart disease (IHD), ischaemic stroke (IS) and peripheral artery disease (PAD),^14^ and (ii) autoimmune disease, composed of eight conditions previously linked to type I interferons (Supplementary Table 4). Secondary outcomes included components of primary outcomes and additional cardiovascular endpoints (Supplementary Table 5). Prevalent chronic conditions (Supplementary Table 6) were defined using a look-back period of up to 5 years^15^ and participants with the outcome prior to their index date were excluded.

### Statistical analyses

We estimated the association between interferon-β therapy duration and outcomes with Cox regressions using time-dependent covariates, stratified by categorical calendar year at index date (four strata) to account for secular trends and handle non-proportional baseline hazards. Interferon-β therapy was modelled using both a never/ever exposure variable (two levels) and a treatment duration variable (continuous). These exposure variables were updated at the beginning of 30-day follow-up intervals and carried forward until the study end.^16^ For primary outcomes, we also modelled treatment duration using a four-level discrete variable instead of a linear term to examine associations per duration strata: 0 to <0.5 (reference), 0.5 to <6.5, 6.5 to <12.5, 12.5 to 22 years. Age-standardized cardiovascular disease rates were calculated to help interpret relative measures of association.

Regressions were adjusted for age (third-order polynomial), sex and socioeconomic status (continuous), season (time-updated, four categories), exposure to other DMTs (one time-updated never/ever exposure variable per DMT except daclizumab and ocrelizumab for which the few users [*n* < 10] were censored upon filling their first prescription) and, for cardiovascular outcomes, hypertension, diabetes mellitus, hyperlipidaemia, obesity, sleep apnoea, chronic kidney disease, depression, epilepsy and Charlson Comorbidity Index. Participants leaving the cohort due to the competing risk of death were censored at the time of death in cause-specific models. Formulation-specific effects were explored for primary outcomes, and glatiramer acetate (negative control exposure) and haemorrhoids (negative control outcome) were modelled to help detect residual confounding and issues related to competing risks.^17^

Four sensitivity analyses were performed to explore potential bias from misclassification of exposure (starting follow-up at index date + 30 days, excluding participants shifted forward) and stroke (exclusion of diagnoses ≤30 days following MS diagnosis, definition from hospital visits only^18^). We also reanalysed the primary cardiovascular outcome after including cardiovascular deaths (i.e. due to IHD, IS or PAD) and different proportions of those censored due to non-cardiovascular deaths as failures to explore potential bias due to competing risks. We report adjusted hazard ratios (HR) with 95% confidence intervals (CI) per five-year increment in interferon-β therapy duration (mean interferon-β therapy duration) and defined statistical significance as *P*-value <0.05. All analyses were conducted on the Secure Research Environment of Population Data BC using RStudio (v1.3).

## Results

**Figure 1:**
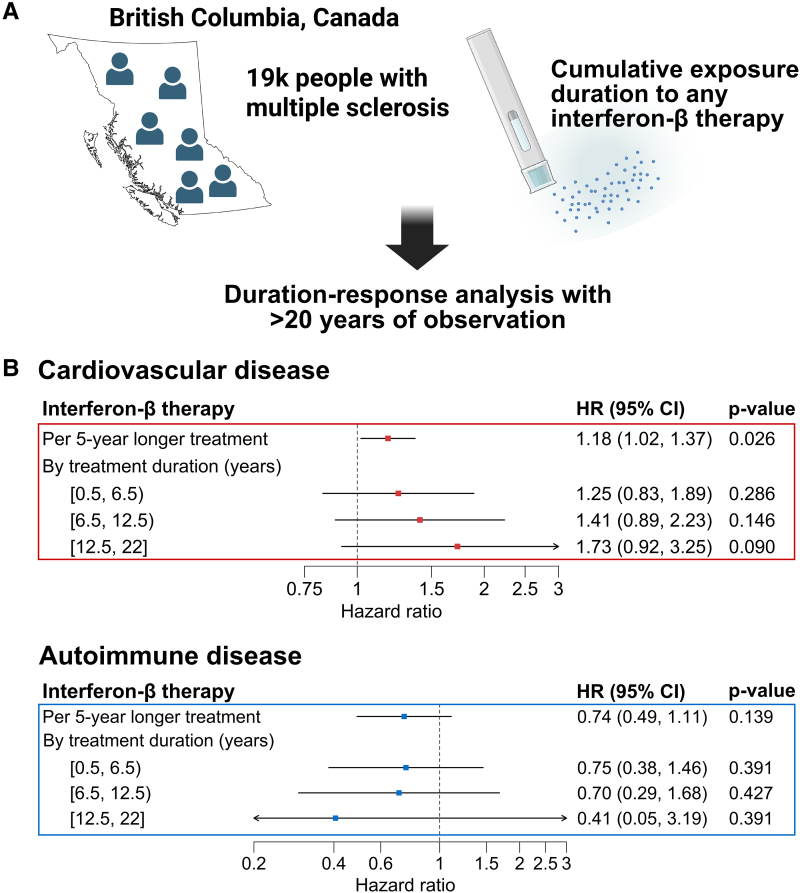
**Longer interferon-β therapy is associated with incident cardiovascular disease but not with autoimmunity in people with MS.** (**A**) Graphical summary of the study design. This population-based cohort used province-wide (British Columbia, Canada) linked administrative health databases for hospital/physician visits and filled prescriptions from all community/hospital outpatient pharmacies. The cumulative exposure duration to interferon-β (any formulation) was calculated using the days supplied. Created in BioRender. Rioux, B. (2025): https://BioRender.com/mqcr35e. (**B**) Associations between longer interferon-β therapy and primary outcomes. Cumulative interferon-β therapy duration was examined as a linear (HR per 5-year increment in treatment duration) and categorical variable (strata of treatment duration in years relative to <0.5 year) and estimates are from fully adjusted Cox regression models (*N* = 19 360 participants in the full cohort). Cardiovascular disease is a composite of ischaemic heart disease, ischaemic stroke and peripheral artery disease, and autoimmune disease is a composite of Addison’s disease, coeliac disease, dermatomyositis/polymyositis, primary biliary cirrhosis, rheumatoid arthritis, Sjogren disease, systemic lupus erythematosus and systemic sclerosis. Axes are on the log10 scale. For intervals, closed and open brackets respectively indicate the endpoint is included and excluded. CI, confidence interval; HR, hazard ratio.

The cohort included 19 360 people with MS followed for a median duration of 11.2 years (Q1-Q3: 5.1–18.7), of whom 3138 (16.2%) were ever treated with an interferon-β (median treatment duration = 3.3 years; Q1–Q3: 1.2–7.2; Fig. 1A). The mean (SD) age at index date was 37.2 (9.7) and 45.9 (13.7) years for the ever and never users, respectively. Participants ever exposed to an interferon-β, as compared to those never exposed, had a lower prevalence of comorbidities and were more frequently exposed to other DMTs (Table 1 and Supplementary Table 7).

**Table 1:** Description of people with MS in the study cohort

Cohort characteristics (at index date unless specified)	Never exposed to interferon-β (*n* = 16 222)	Ever exposed to interferon-β (*n* = 3138)
**Demographics**		
Age, mean (SD)	45.9 (13.7)	37.2 (9.7)
Male sex, *n* (%)	4595 (28.3)	825 (26.3)
**Charlson Comorbidity Index, *n* (%)**		
0	13 225 (81.5)	2861 (91.2)
1	1470 (9.1)	156 (5.0)
2	771 (4.8)	82 (2.6)
≥3	756 (4.7)	39 (1.2)
**Comorbidities, *n* (%)**		
Chronic kidney disease	88 (0.5)	9 (0.3)
Depression	4546 (28.0)	811 (25.8)
Diabetes mellitus	798 (4.9)	65 (2.1)
Epilepsy	343 (2.1)	40 (1.3)
Hyperlipidaemia	942 (5.8)	72 (2.3)
Hypertension	2254 (13.9)	183 (5.8)
Obesity	666 (4.1)	140 (4.5)
**Use of non-interferon-β DMT (from entry to exit date), *n* (%)**		
Any	2012 (12.4)	1864 (59.4)
Glatiramer acetate	979 (6.0)	735 (23.4)
Fingolimod	147 (0.9)	274 (8.7)
Dimethyl fumarate	413 (2.5)	339 (10.8)
Teriflunomide	286 (1.8)	232 (7.4)
Alemtuzumab	97 (0.6)	82 (2.6)
Natalizumab	88 (0.5)	197 (6.3)

There were ≤5 ever users of daclizumab or ocrelizumab in each group (not shown). DMT, disease-modifying therapy.

**Figure 2:**
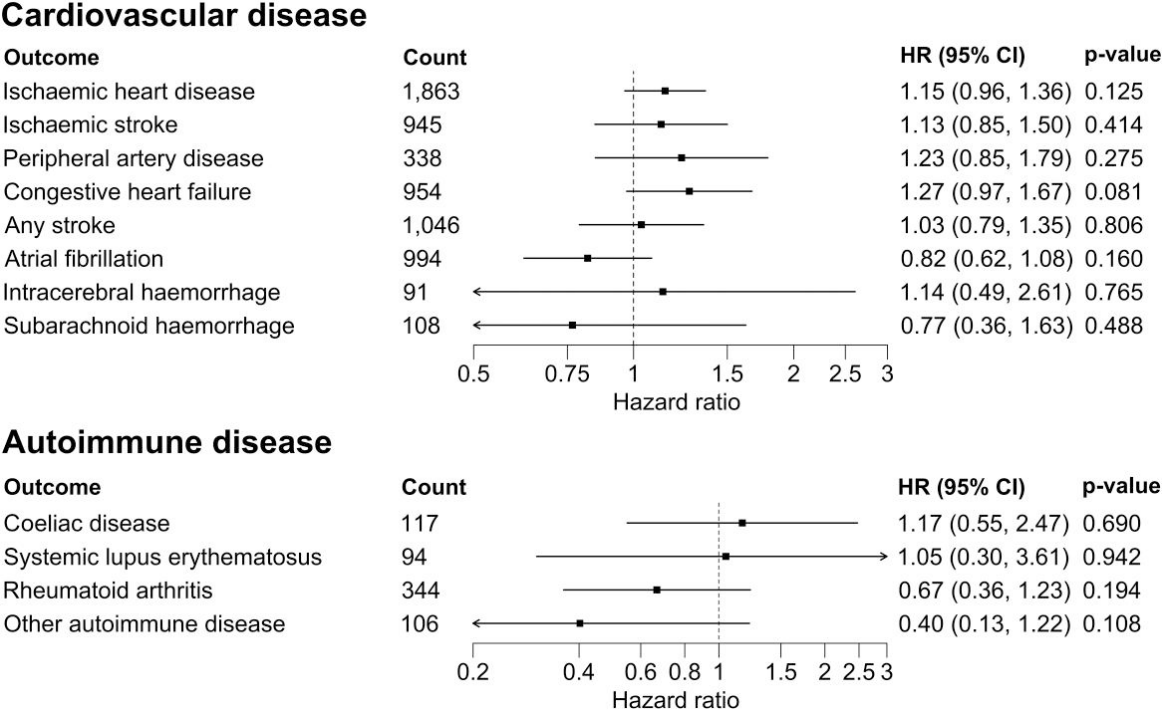
**Association of longer interferon-β therapy duration with secondary cardiovascular and autoimmune outcomes in people with MS.** Cumulative interferon-β therapy duration examined using a linear variable and associations reported per 5-year increment in treatment duration from fully adjusted Cox regression models (*N* = 19 360 participants in the full cohort). ‘Other autoimmune disease’ includes Addison’s disease, dermatomyositis/polymyositis, primary biliary cirrhosis, Sjogren disease and systemic sclerosis (grouped to preserve statistical power). Axes are on the log10 scale. CI, confidence interval; HR, hazard ratio.

Longer interferon-β therapy was associated with a higher incidence of cardiovascular disease (per 5-year longer treatment: HR = 1.18; 95% CI: 1.02, 1.37; *P* = 0.026) but was not associated with autoimmunity (per 5-year longer treatment: HR = 0.74; 95% CI: 0.49, 1.11; *P* = 0.139; Fig. 1B and Supplementary Table 8). The age-standardized cardiovascular disease rate was increased by 3.6 per 1000 person-years in the highest exposure strata (i.e. 12.5 to 22 years on treatment) as compared to the projected rate if unexposed (13.0 versus 9.4 per 1000 person-years; Supplementary Fig. 2). Hazard ratios for IHD, IS and PAD were all >1 and the direction of association for congestive heart failure was consistent with that of IHD (Fig. 2). Rebif®, Betaseron® and Avonex® had HR point estimates >1 for cardiovascular disease risk, but Rebif® had the strongest association and appeared to drive the signal observed across any interferon-β (HR = 1.40; 95% CI: 1.12, 1.76; *P* = 0.003; Table 2). Their association with autoimmunity was less consistent and appeared lower for Betaseron®. Longer interferon-β exposure was not associated with the risk of haemorrhoids (HR = 1.07; 95% CI: 0.88, 1.32; *P* = 0.489), while longer glatiramer acetate treatment was not associated with cardiovascular (HR = 0.77; 95% CI: 0.56, 1.08; *P* = 0.134) or autoimmune disease (HR = 1.51; 95% CI: 0.92, 2.46; *P* = 0.101). Results were comparable in sensitivity analyses (Supplementary Fig. 3) and after accounting for potential bias due to the competing risk of death (Supplementary Table 9).

**Table 2:** Distribution and association with primary outcomes of interferon-β medications used in the cohort

Interferon-β subtype	Interferon-β medication	Ever users, *n* (%)^a^	Therapy duration, total person-years (median; Q1-Q3)	Cardiovascular disease		Autoimmune disease	
				HR (95% CI)	*P*-value	HR (95% CI)	*P*-value
Any		3138 (100)	15 065 (3.3; 1.2–7.2)	**1.18 (1.02, 1.37)**	**0.026**	0.74 (0.49, 1.11)	0.139
Interferon-β−1a	Any	2381 (75.9)	9847 (2.6; 0.9–6.1)	**1.33 (1.08, 1.62)**	**0**.**006**	0.99 (0.61, 1.62)	0.980
	Rebif®	1699 (54.1)	6756 (2.5; 0.8–5.8)	**1.40 (1.12, 1.76)**	**0**.**003**	0.60 (0.29, 1.25)	0.171
	Avonex®	871 (27.8)	3089 (2.0; 0.8–5.1)	1.04 (0.69, 1.56)	0.853	1.76 (0.90, 3.42)	0.097
Interferon-β−1b	Any	1124 (35.8)	5218 (2.8; 1.0–7.0)	1.06 (0.86, 1.31)	0.567	**0.39** (**0.18, 0.85)**	**0**.**018**
	Betaseron®	1106 (35.2)	5171 (2.8; 1.0–7.1)	1.08 (0.87, 1.33)	0.479	**0.39** (**0.18, 0.84)**	**0**.**017**
	Extavia®	29 (0.9)	47 (1.0; 0.3–1.6)	NA^b^		NA^b^	

^a^Denominators refer to users of any interferon-β medications.

^b^Extavia® was not modelled due to the small number of users. Plegridy® is not shown due to small non-zero cell sizes (<5 users) as per data privacy agreements. Results are from fully adjusted models. For interferon-β subtypes, a never/ever exposure variable for any other interferon-β medications was added to account for the effect of other interferon-β medications used during follow-up (e.g. for Rebif®, the binary term indicates whether participants were ever exposed to an interferon-β other than Rebif®). Bold characters indicate *P* < 0.05. CI, confidence interval; HR, hazard ratio.

## Discussion

In this population-based study of people with MS, we observed a statistically significant but modest increase in cardiovascular disease risk associated with longer interferon-β treatment, consistent with a duration-response relationship. Hazard ratio point estimates were increased across cardiovascular diseases due to atherosclerosis, including IHD, IS, PAD and congestive heart failure (most often due to IHD),^19^ although these secondary outcomes had lower event counts leading to wider CIs that crossed the null value. Rebif®, often considered the most ‘potent’ interferon-β with a high dose and frequency administration regimen,^20,21^ appeared to confer the strongest risk of cardiovascular disease across interferon-β medications, supporting an exposure-response relationship between interferon-β and cardiovascular disease.

Interferon-β is a pro-atherosclerotic cytokine and essential modulator of atherosclerosis.^14,22^ In atherosclerosis-susceptible mice, interferon-β treatment promotes endothelial adhesion of macrophages through CCL5-CCR5-dependent mechanisms, increases macrophage accumulation in lesions and enhances plaque development without affecting plasma cholesterol levels, a process that can be halted by altering interferon-β receptors in myeloid cells.^1^ Type I interferon signalling is significantly upregulated in ruptured versus unruptured atherosclerotic plaques, while interferon-β-induced chemotactic factors such as CCL5 and CCR5 are upregulated in ruptured plaques.^1^ Likewise, depletion of plasmacytoid dendritic cells (a major source of circulating type I interferons) in mouse models of atherosclerosis reduces plaque formation and macrophage content in atherosclerotic lesions, while promoting plaque stability through increased collagen content.^23^ Taken together, these studies provide experimental evidence supporting a mechanistically plausible link between a sustained elevation of circulating interferon-β and atherosclerotic disease.

High circulating type I interferons is a hallmark of ‘interferonopathic’ autoimmune diseases such as systemic lupus erythematosus.^3^ We did not, however, identify an increased risk of autoimmunity in our study, which may partly be explained by three factors. First, interferon-related autoimmune disorders are primarily associated with interferon-α which, despite having substantial structural homology and sharing a common heterodimeric cell-surface receptor (IFNAR1/IFNAR2) with interferon-β, has distinct biological triggers and downstream effects.^24^ Second, interferon-β has immunomodulatory properties which, unlike interferon-α, may play against the emergence of comorbid autoimmunity in MS.^25^ Finally, exogenous interferon-β used in MS may not fully recapitulate the conditions endogenous type I interferons need to induce autoimmunity, such as an appropriate timing of exposure, biological compartment, cell source and concentration.^24^

Adverse cardiovascular events associated with longer interferon-β therapy are not listed by major medicines regulatory agencies (Supplementary Table 10). This new signal has direct clinical implications for physicians and people with MS. First, this potential long-term risk warrants paying closer attention to primary cardiovascular prevention strategies (e.g. screening and treating high blood pressure) in prior and current interferon-β users. This is especially relevant as interferon-β remains commonly used in older MS patients (42% of participants randomized in the recent DISCOMS trial assessing DMT discontinuation in the USA),^26^ a population already at higher risk of cardiovascular disease, and because patients with MS have a higher prevalence yet poorer management of cardiometabolic risk factors as compared to the general population.^27^ Second, clinicians may want to include this safety signal in their shared decision-making discussion when comes the time to initiate, modify or stop a DMT in patients at high risk or with established cardiovascular disease. The clinical benefits of interferon-β in MS, however, are well established^28^ and likely outweigh modest increases in cardiovascular disease risk in most people. This consideration is therefore most pertinent when discontinuing DMTs is being considered,^26^ especially in people who have been on interferon-β therapy for an extended period.

The strengths of this study include a systematic linkage of filled prescriptions contributing to the overall accuracy of inferred DMT exposures and the long follow-up offering a unique opportunity to capture long-term adverse events. Our study, however, has some limitations. First, our analyses have low statistical power overall due to the narrow distribution of treatment durations and, despite the large sample size, the small proportion ever exposed to an interferon-β. Second, we cannot rule out some degree of bias away from the null due to the competing risk of death, but this is unlikely to have driven the risk of cardiovascular disease or alter conclusions. Sensitivity analyses treating deaths as failures yielded similar results, and the two negative controls subject to similar patterns of competing risk and confounding were neutral. Third, we cannot rule out residual confounding by indication or from unmeasured potentially confounding variables such as an individual’s body mass index, smoking or disability status (e.g. Expanded Disability Status Scale score). Patterns of confounding may also differ by formulation (e.g. Rebif® versus others) in sensitivity analyses which, combined with their smaller sample sizes, should be interpreted with caution. However, given the overall ‘healthier’ group of participants treated with an interferon-β, such bias is most likely to have pulled the estimates towards a neutral association for cardiovascular disease. Fourth, we tested a focused hypothesis and examining the association of long-term interferon-β treatment with a wider range of adverse events may be relevant in future studies.

In summary, we identified an increased risk of cardiovascular disease associated with longer interferon-β therapy in a large population-based drug safety study. Although this association fulfils several criteria for causality (temporality, exposure-response gradient, experimental evidence and biological plausibility), its broad implications for clinicians and patients call for replication. In the meantime, clinicians should seek to optimize the prevention of cardiovascular disease in people with MS and may want to include this potential risk in their shared decision-making discussion.

## Supplementary Material

fcaf363_Supplementary_Data

## Data Availability

Access to data provided by the Data Steward(s) is subject to approval, but can be requested for research projects through the Data Steward(s) or their designated service providers. The following data sets were used in this study: Discharge Abstract Database, Medical Services Plan Payment Information File, PharmaNet, Central Demographics File and Vital Statistics. Readers can find further information regarding these data sets by visiting the Population Data BC project webpage at: https://my.popdata.bc.ca/project_listings/18-120/collection_approval_dates. All inferences, opinions and conclusions drawn in this publication are those of the author(s), and do not reflect the opinions or policies of the Data Steward(s). Code used for this study can be found at https://github.com/barioux/ifn_drug_safety.git.
