# Supplementary material for: Risk of cardiovascular and autoimmune disease in people with multiple sclerosis on long-term interferon-β therapy
Source: Brain Commun. 2025 Sep 22;7(5):fcaf363. doi: 10.1093/braincomms/fcaf363 (PMC12620613; doi:10.1093/braincomms/fcaf363)
Supplement: fcaf363_Supplementary_Data [file fcaf363_Supplementary_Data.docx]

**SUPPLEMENTARY MATERIAL**

Risk of cardiovascular and autoimmune disease in people with multiple sclerosis on long-term interferon-β therapy

**Authors and affiliations**

Bastien Rioux, MD, MSc^1,2^, Feng Zhu, PhD^3^, Huah Shin Ng, PhD^4,5^, Yinshan Zhao, PhD^3^, Thomas M Caparrotta, PhD, MRCP^1,6^, William N Whiteley, PhD, FRCP^1,7^, David P.J. Hunt, PhD, FRCP^1,2^*, Helen Tremlett, PhD^3^*

^1^ Centre for Clinical Brain Sciences, University of Edinburgh, Edinburgh, United Kingdom

^2^ UK Dementia Research Institute at Edinburgh, Edinburgh, United Kingdom

^3^ Department of Medicine (Neurology) and the Djavad Mowafaghian Centre for Brain Health, University of British Columbia, Vancouver, British Columbia, Canada

^4^ Flinders Health and Medical Research Institute, College of Medicine and Public Health, Flinders University, Adelaide, Australia

^5^ SA Pharmacy, SA Health, Adelaide, Australia

^6^ Department of Clinical Pharmacology, NHS Lothian, Edinburgh, United Kingdom

^7^ British Heart Foundation Data Science Centre, Health Data Research UK, London, United Kingdom

* David P.J. Hunt and Helen Tremlett contributed equally to this work

**Table of Contents**

[SUPPLEMENTARY TABLES 3](#_Toc204334348)

[Supplementary Table 1. The RECORD statement for pharmacoepidemiology (RECORD-PE) checklist of items. 3](#_Toc204334349)

[Supplementary Table 2. Disease-modifying therapies approved by Health Canada for the treatment of multiple sclerosis over the study period. 11](#_Toc204334350)

[Supplementary Table 3. Multiple sclerosis and other central nervous system demyelinating diagnostic codes. 12](#_Toc204334351)

[Supplementary Table 4. Summary of evidence supporting a link between autoimmune diseases included as outcomes and type I interferons. 13](#_Toc204334352)

[Supplementary Table 5. Case definitions and counts for outcomes in the cohort. 14](#_Toc204334353)

[Supplementary Table 6. Case definitions and counts for covariates in the cohort. 24](#_Toc204334354)

[Supplementary Table 7. Description of people with multiple sclerosis in the study cohort (continued). 28](#_Toc204334355)

[Supplementary Table 8. Hazard ratios for clinical outcomes with unadjusted and adjusted models. 29](#_Toc204334356)

[Supplementary Table 9. Sensitivity analyses for the competing risk of death in the primary cardiovascular model (fully adjusted model). 30](#_Toc204334357)

[Supplementary Table 10. Warnings and precautions with recommended actions for interferon-β formulations used in the cohort. 31](#_Toc204334358)

[SUPPLEMENTARY FIGURES 34](#_Toc204334359)

[Supplementary Fig. 1. Graphical summary of the study follow-up. 34](#_Toc204334360)

[Supplementary Fig. 2. Age-standardised cardiovascular disease rates by treatment group. 35](#_Toc204334361)

[Supplementary Fig. 3. Forest plots of adjusted hazard ratios for cardiovascular and autoimmune diseases after (A) starting follow-up at index date plus 30 days, (B) removing participants with an index date shifted forward, (C) removing strokes occurring within the first 30 days of follow-up, and (D) removing strokes only defined through physician visits. 36](#_Toc204334362)

[SUPPLEMENTARY REFERENCES 37](#_Toc204334363)

# SUPPLEMENTARY TABLES

## Supplementary Table 1. The RECORD statement for pharmacoepidemiology (RECORD-PE) checklist of items.

| **Item**  **No** | **STROBE items** | **RECORD items** | **RECORD-PE items** | **Section** |
| --- | --- | --- | --- | --- |
| **Title and abstract** | | | | |
| 1 | (a) Indicate the study’s design with a commonly used term in the title or the abstract.  (b) Provide in the abstract an informative and balanced summary of what was done and what was found. | 1.1: The type of data used should be specified in the title or abstract. When possible, the name of the databases used should be included. 1.2: If applicable, the geographical region and timeframe within which the study took place should be reported in the title or abstract. 1.3: If linkage between databases was conducted for the study, this should be clearly stated in the title or abstract. | — | **Title, abstract** |
| **Introduction** | | | | |
| Background rationale | | | | |
| 2 | Explain the scientific background and rationale for the investigation being reported. | — | — | **Introduction, paragraphs 1-2** |
| Objectives | | | | |
| 3 | State specific objectives, including any prespecified hypotheses. | — | — | **Introduction, paragraph 2** |
| **Methods** | | | | |
| Study design | | | | |
| 4 | Present key elements of study design early in the paper. | — | 4.a: Include details of the specific study design (and its features) and report the use of multiple designs if used.  4.b: The use of a diagram(s) is  recommended to illustrate key aspects of the study design(s), including exposure, washout, lag and observation periods, and covariate definitions as relevant. | **Methods – Data sources and study population** |
| Setting | | | | |
| 5 | Describe the setting, locations, and relevant dates, including periods of recruitment, exposure, follow-up, and data collection. | — | — | **Methods – Data sources and study population, Figure 1** |
| Participants | | | | |
| 6 | (a) Cohort study—give the eligibility criteria, and the sources and methods of selection of participants. Describe methods of follow-up. Case-control study—give the eligibility criteria, and the sources and methods of case ascertainment and control election. Give the rationale for the choice of cases and controls. Cross sectional study—give the eligibility criteria, and the sources and methods of selection of participants.  (b) Cohort study—for matched studies, give matching criteria and number of exposed and unexposed. Case-control study—for matched studies, give matching criteria and the number of controls per case. | 6.1: The methods of study  population selection (such as codes or algorithms used to identify participants) should be listed in detail. If this is not possible, an explanation should be provided. 6.2: Any validation studies of the codes or algorithms used to select the population should be  referenced. If validation was  conducted for this study and not published elsewhere, detailed methods and results should be provided.  6.3: If the study involved linkage of databases, consider use of a flow diagram or other graphical display to demonstrate the data linkage process, including the number of individuals with linked data at each stage. | 6.1.a: Describe the study entry criteria and the order in which these criteria were applied to identify the study population. Specify whether only users with a specific indication were included and whether patients were allowed to enter the study population once or if multiple entries were permitted. See explanatory document for guidance related to matched designs. | **Methods – Data sources and study population** |

| Variables | | | |  |
| --- | --- | --- | --- | --- |
| 7 | Clearly define all outcomes, exposures, predictors, potential confounders, and effect modifiers. Give diagnostic criteria, if applicable. | 7.1: A complete list of codes and algorithms used to classify  exposures, outcomes, confounders, and effect modifiers should be provided. If these cannot be  reported, an explanation should be provided. | 7.1.a: Describe how the drug exposure definition was developed.  7.1.b: Specify the data sources from which drug exposure information for individuals was obtained.  7.1.c: Describe the time window(s) during which an individual is considered exposed to the drug(s). The rationale for selecting a particular time window should be provided. The extent of potential left truncation or left censoring should be specified.  7.1.d: Justify how events are attributed to current, prior, ever, or cumulative drug exposure.  7.1.e: When examining drug dose and risk attribution, describe how current, historical or time on therapy are  considered.  7.1.f: Use of any comparator groups should be outlined and justified.  7.1.g: Outline the approach used to handle individuals with more than one relevant drug exposure during the study period. | **Methods – Definition of exposures, outcomes and covariates** |
| Data sources/measurement | | | |  |
| 8 | For each variable of interest, give sources of data and details of methods of  assessment (measurement). Describe comparability of assessment methods if there is more than one group. | — | 8.a: Describe the healthcare system and mechanisms for generating the drug exposure records. Specify the care setting in which the drug(s) of interest was prescribed. | **Methods – Definition of exposures, outcomes and covariates** |
| Bias | | | |  |
| 9 | Describe any efforts to address potential sources of bias. | — | — | **Methods – Statistical analyses** |
| Study size | | | |  |
| 10 | Explain how the study size was arrived at. | — | — | **Methods – Data source and study population** |
| Quantitative variables | | | |  |
| 11 | Explain how quantitative variables were handled in the analyses. If applicable, describe which groupings were chosen, and why. | — | — | **Methods – Statistical analyses** |
| Statistical methods | | | |  |
| 12 | (a) Describe all statistical methods, including those used to control for confounding.  (b) Describe any methods used to examine subgroups and interactions.  (c) Explain how missing data were addressed.  (d) Cohort study—if applicable, explain how loss to follow-up was addressed. Case-control study—if applicable, explain how matching of cases and controls was addressed. Cross sectional study—if applicable, describe analytical methods taking account of sampling strategy. (e) Describe any sensitivity analyses. | — | 12.1.a: Describe the methods used to evaluate whether the assumptions have been met.  12.1.b: Describe and justify the use of multiple designs, design features, or analytical approaches. | **Methods – Statistical analyses** |

| Data access and cleaning methods | | | | |
| --- | --- | --- | --- | --- |
| 12 | — | 12.1: Authors should describe the extent to which the investigators had access to the database  population used to create the study population.  12.2: Authors should provide information on the data cleaning methods used in the study. | — | **Methods – Definition of exposures, outcomes and covariates** |
| Linkage | | | | |
| 12 | — | 12.3: State whether the study included person level, institutional level, or other data linkage across two or more databases. The  methods of linkage and methods of linkage quality evaluation should be provided. | — | **Methods – Data sources and study population** |
| **Results** | | | | |
| Participants | | | | |
| 13 | (a) Report the numbers of individuals at each stage of the study (eg, numbers potentially eligible, examined for  eligibility, confirmed eligible, included in the study, completing follow-up, and analysed).  (b) Give reasons for non-participation at each stage.  (c) Consider use of a flow diagram. | 13.1: Describe in detail the  selection of the individuals included in the study (that is, study  population selection) including filtering based on data quality, data availability, and linkage. The selection of included individuals can be described in the text or by means of the study flow diagram. | — | **Results, paragraph 1** |
| Descriptive data | | | | |
| 14 | (a) Give characteristics of study  participants (eg, demographic, clinical, social) and information on exposures and potential confounders.  (b) Indicate the number of participants with missing data for each variable of interest.  (c) Cohort study—summarise follow-up time (eg, average and total amount). | — | — | **Results, paragraph 1, Table 1** |
| Outcome data | | | | |
| 15 | Cohort study—report numbers of outcome events or summary measures over time. Case-control study—report numbers in each exposure category, or summary measures of exposure. Cross sectional study—report numbers of outcome events or summary measures. | — | — | **Results, paragraph 2, Figures 1-2** |
| Main results | | | | |
| 16 | (a) Give unadjusted estimates and, if applicable, confounder adjusted estimates and their precision (eg, 95% CI). Make clear which confounders were adjusted for and why they were included. (b) Report category boundaries when continuous variables are categorised. | — | — | **Results, paragraph 2** |

|  | (c) If relevant, consider translating estimates of relative risk into absolute risk for a meaningful time period. |  |  |  |
| --- | --- | --- | --- | --- |
| Other analyses | | | | |
| 17 | Report other analyses done—eg, analyses of subgroups and interactions, and sensitivity analyses. | — | — | **Results, paragraph 2** |
| **Discussion** | | | | |
| Key results | | | | |
| 18 | Summarise key results with reference to study objectives. | — | — | **Discussion, paragraph 1** |
| Limitations | | | | |
| 19 | Discuss limitations of the study, taking into account sources of potential bias or imprecision. Discuss both direction and magnitude of any potential bias. | 19.1: Discuss the implications of using data that were not created or collected to answer the specific research question(s). Include discussion of misclassification bias, unmeasured confounding, missing data, and changing eligibility over time, as they pertain to the study being reported. | 19.1.a: Describe the degree to which the chosen database(s) adequately captures the drug exposure(s) of interest. | **Discussion, paragraph 5** |
| Interpretation | | | | |
| 20 | Give a cautious overall interpretation of results considering objectives, limitations, multiplicity of analyses, results from similar studies, and other relevant  evidence. | — | 20.a: Discuss the potential for  confounding by indication,  contraindication or disease severity or selection bias (healthy adherer/sick stopper) as alternative explanations for the study findings when relevant. **[A: Original text indicated this item was RECORD (ie, not RECORD-PE)?]** | **Discussion, paragraph 4** |
| Generalisability | | | | |
| 21 | Discuss the generalisability (external validity) of the study results. | — | — | **Discussion, paragraph 5** |
| **Other information** | | | | |
| Funding | | | | |
| 22 | Give the source of funding and the role of the funders for the present study and, if applicable, for the original study on which the present article is based. | — | — | **Funding statement** |
| Accessibility of protocol, raw data, and programming code | | | | |
| 22 | — | 22.1: Authors should provide information on how to access any supplemental information such as the study protocol, raw data, or programming code. | — | **Data sharing statement** |

## Supplementary Table 2. Disease-modifying therapies approved by Health Canada for the treatment of multiple sclerosis over the study period.

| **Disease-modifying therapy** | **Brand name** | **Health Canada approval date** |
| --- | --- | --- |
| Interferon-β-1b | Betaseron^®^ | July 1995 |
|  | Extavia^®^ | January 2010 |
| Interferon-β-1a | Rebif^®^ | February 1998 |
|  | Avonex^®^ | April 1998 |
|  | Plegridy^®^ | August 2015 |
| Glatiramer acetate | Copaxone^®^ | October 1997 |
| Natalizumab | Tysabri^®^ | October 2006 |
| Fingolimod | Gilenya^®^ | March 2011 |
| Dimethyl fumarate | Tecfidera^®^ | Avril 2013 |
| Teriflunomide | Aubagio^®^ | November 2013 |
| Alemtuzumab | Lemtrada^®^ | December 2013 |
| Daclizumab | Zinbryta^®^ | December 2016 (withdrawn in 2018 due to risk of encephalitis) |
| Ocrelizumab | Ocrevus^®^ | September 2017 |

## Supplementary Table 3. Multiple sclerosis and other central nervous system demyelinating diagnostic codes.

| **Diagnosis** | **ICD v9** | **ICD v10** |
| --- | --- | --- |
| Multiple sclerosis | 340 | G35 |
| Optic neuritis | 377.3 | H46 |
| Acute transverse myelitis | 323.82  341.2 | G37.3 |
| Acute disseminated encephalomyelitis | 323 | G36.9 |
| Demyelinating disease of central nervous system, unspecified | 341.9 | G37.8 |
| Other acute disseminated demyelination | NA | G36 |
| Neuromyelitis optica | 341.0 | G36.0 |

Abbreviations: ICD, International Classification of Diseases.

## Supplementary Table 4. Summary of evidence supporting a link between autoimmune diseases included as outcomes and type I interferons.

| **Outcome** | **Mechanistic link with type I interferons** | **Peripheral type I interferon signature** | **Association with type I interferon therapy** | **References** |
| --- | --- | --- | --- | --- |
| Addison’s disease | Possible | Yes | Not reported, but worsening of disease | 1,2 |
| Coeliac disease | Possible | Unclear; raised ISGs in intestinal mucosa | Yes | 3,4 |
| Dermatomyositis/  polymyositis | Yes | Yes | Yes | 5-8 |
| Systemic lupus erythematosus | Yes | Yes | Yes | 9,10 |
| Primary biliary cirrhosis | Possible | Yes | Yes | 11-14 |
|  |  |  |  |  |
| Rheumatoid arthritis | Yes | Yes | Yes | 15,16 |
| Sjogren disease | Yes | Yes | Yes | 17,18 |
| Systemic sclerosis | Yes | Yes | Yes | 19,20 |

We aimed to test a specific hypothesis that chronic exposure to interferon-β increases the risk of autoimmune disease. Conditions that we included had some evidence of peripheral type I interferon signatures and a plausible mechanistic link with chronically raised type I interferons in blood. Autoimmune thyroid diseases were not included as these are most often seen early after treatment onset and have weaker evidence supporting a duration-response pattern. Abbreviations: ISG, interferon-stimulated gene.

## Supplementary Table 5. Case definitions and counts for outcomes in the cohort.

| **Condition/definition** | **ICD codes** | | **Total count from sources** | **Sources (%)** | **Events before entry date** | **Events between entry and exit date** |
| --- | --- | --- | --- | --- | --- | --- |
|  | **v9** | **v10** |  |  |  |  |
| **Cardiovascular disease: primary outcome** | | | | | | |
| **Atherosclerotic disease** | Any diagnosis of ischaemic heart disease, ischaemic stroke or peripheral artery disease (as per definitions below). | | 4,319 | P: 58.9;  H&P: 33.4;  H: 7.8. | 1,635 | 2,564 |
| **Cardiovascular disease: secondary outcomes** | | | | |  |  |
| **Atrial fibrillation**  **≥1 hospital discharge diagnosis or ≥4 diagnostic billing codes on separate days within 1 year, any position**  Adapted^21^: ≥1 hospital discharge diagnosis or ≥4 diagnostic billing code within 1 year in Ontario; Se: 70.8%, PPV: 70.8%; codes used: 427, I48. | 427 (cardiac dysrhythmias)^21^ | I48 (atrial fibrillation and flutter)^21^ | 1,417 | P: 40.0;  H&P: 30.0;  H: 30.0. | 369 | 994 |
| **Any stroke**  **≥1 hospital discharge diagnosis or ≥2 diagnostic billing codes on separate days within 1 year, any position**  Adapted^22^: ≥1 hospital discharge diagnosis or ≥2 diagnostic billing codes within 1 year in Ontario for stroke or TIA; Se: 68.0-73.3%, PPV: 66.2-85.7%. | 430 (subarachnoid haemorrhage), 431 (intracerebral haemorrhage), 433 (occlusion and stenosis of precerebral arteries), 434 (occlusion of cerebral arteries), 436 (acute, but ill-defined, cerebrovascular disease), 362.3 (retinal vascular occlusion); adapted^22^ | I60 (subarachnoid haemorrhage), I61 (intracerebral haemorrhage), I63 (cerebral infarction), I64 (stroke, not specified as haemorrhage or infarction), H34.0 (transient retinal artery occlusion), H34.1 (central retinal artery occlusion), H34.2 (other retinal artery occlusions); adapted^22^ | 1,701 | P: 59.6;  H&P: 28.0;  H: 12.4. | 617 | 1,046 |
| **Congestive heart** **failure**  **≥1 hospital discharge diagnosis or ≥2 diagnostic billing codes on separate days within 1 year, any position**  Adapted^23^: ≥1 hospital discharge diagnosis or ≥2 diagnostic billing codes within 1 year in Ontario; Se: 84.8%, PPV: 55.6%; codes used: 428, I50.0, I50.1, I50.9. | 428 (heart failure)^23^ | I50 (heart failure)^23^ | 1,180 | P: 46.4;  H&P: 34.9;  H: 18.7. | 187 | 954 |
| **Intracerebral haemorrhage**  **≥1 hospital discharge diagnosis or ≥2 diagnostic billing codes on separate days within 1 year, any position**  Adapted^22^: ≥1 hospital discharge diagnosis or ≥2 diagnostic billing codes within 1 year in Ontario for stroke or TIA; Se: 68.0-73.3%, PPV: 66.2-85.7%. | 431 (intracerebral haemorrhage)^22^ | I61 (intracerebral haemorrhage)^22^ | 131 | P: 43.5;  H: 34.4;  H&P: 22.1. | 37 | 91 |
| **Ischaemic heart disease**  **≥1 hospital discharge diagnosis or ≥2 diagnostic billing codes on separate days within 3 years, any position**  Adapted^24^: ≥1 hospital discharge diagnosis (I20-I25) or ≥2 diagnostic billing codes (410, 412, 413) within 3 years in Ontario; Se: 78.2%, PPV: 74.7%. | 410 (acute myocardial infarction), 411 (other acute and subacute forms of ischemic heart disease), 412 (old myocardial infarction), 413 (angina pectoris), 414 (other forms of chronic ischemic heart disease); adapted^24^ | I20 (angina pectoris), I21 (acute myocardial infarction), I22 (subsequent myocardial infarction), I23 (certain current complications following acute myocardial infarction), I24 (other acute ischaemic heart disease), I25 (chronic ischaemic heart disease)^24^ | 3,091 | P: 60.0;  H&P: 33.4;  H: 6.6. | 1,140 | 1,863 |
| **Ischaemic stroke**  **≥1 hospital discharge diagnosis or ≥2 diagnostic billing codes on separate days within 1 year, any position**  Adapted^22^: ≥1 hospital discharge diagnosis or ≥2 diagnostic billing codes within 1 year in Ontario for stroke or TIA; Se: 68.0-73.3%, PPV: 66.2-85.7%.  TIA not included due to higher misclassification potential with MS symptoms and other stroke mimics in people with MS. | 433 (occlusion and stenosis of precerebral arteries), 434 (occlusion of cerebral arteries), 436 (acute, but ill-defined, cerebrovascular disease), 362.3 (retinal vascular occlusion); adapted^22^ | I63 (cerebral infarction), I64 (stroke, not specified as haemorrhage or infarction), H34.0 (transient retinal artery occlusion), H34.1 (central retinal artery occlusion), H34.2 (other retinal artery occlusions); adapted^22^ | 1,553 | P: 62.3;  H&P: 26.0;  H: 11.7. | 572 | 945 |
| **Peripheral artery disease**  **≥1 hospital discharge diagnosis or diagnostic billing code, any position**  Adapted^25^: ≥1 hospital discharge diagnosis (I73.9, I79.2) or diagnostic billing code (443.9) in Alberta; Se: 8.5-10.2%, PPV: 36.4-64.3%. | 440.0 (atherosclerosis of aorta), 440.2 (atherosclerosis of native arteries of the extremities), 440.3 (atherosclerosis of bypass graft of the extremities), 440.4 (chronic total occlusion of artery of the extremities), 443.9 (peripheral vascular disease, unspecified); adapted^25,26^ | I70.0 (atherosclerosis of aorta), I70.2 (atherosclerosis of arteries of extremities), I73.9 (peripheral vascular disease, unspecified), I79.2 (peripheral angiopathy in diseases classified elsewhere); adapted^25,26^ | 437 | P: 54.0;  H: 33.9;  H&P: 12.1. | 80 | 338 |
| **Subarachnoid haemorrhage**  **≥1 hospital discharge diagnosis or ≥2 diagnostic billing codes on separate days within 1 year, any position**  Adapted^22^: ≥1 hospital discharge diagnosis or ≥2 diagnostic billing codes within 1 year in Ontario for stroke or TIA; Se: 68.0-73.3%, PPV: 66.2-85.7%. | 430 (subarachnoid haemorrhage)^22^ | I60 (subarachnoid haemorrhage)^22^ | 148 | P: 60.8;  H: 23.0;  H&P: 16.2. | 39 | 108 |
| **Autoimmune disease: primary outcome** | | | | | | |
| **Autoimmune disease linked to type I interferons** | Any diagnosis of Addison’s disease, coeliac disease, dermatomyositis/polymyositis, systemic lupus erythematosus, primary biliary cirrhosis, rheumatoid arthritis, Sjogren disease or systemic sclerosis (as per definitions below). | | 1,182 | P: 72.2;  H: 14.0;  H&P: 13.9. | 567 | 580 |
| **Autoimmune disease: secondary outcomes^a^** | | | | | | |
| **Addison’s disease**  **≥1 hospital discharge diagnosis or ≥2 diagnostic billing codes on separate days, any position**  Adapted^27^ | 255.4 (corticoadrenal insufficiency); adapted^27^ | E27.1 (primary adrenocortical insufficiency), E27.2 (addisonian crisis); adapted^27^ | 31 | H: 54.8;  P: n<5;  H&P: n<5. | 9 | 21 |
| **Coeliac disease**  **≥1 hospital discharge diagnosis or ≥2 diagnostic billing codes on separate days, any position**  Adapted^28^: ≥1 hospital discharge diagnosis (K90.0) or ≥2 diagnostic billing codes (579) in Manitoba; Se: 84%, PPV: 80%. | 579 (intestinal malabsorption)^28^ | K90.0 (coeliac disease); adapted^28^ | 208 | H: 42.8;  P: 38.5;  H&P: 18.8. | 86 | 117 |
| **Dermatomyositis/Polymyositis**  **≥1 hospital discharge diagnosis or ≥2 diagnostic billing codes on separate days, any position**  Adapted^29^: ≥1 hospital discharge diagnosis (710.3, 710.4) or ≥2 diagnostic billing codes ≥8 weeks apart in 2 years (or ≥1 if made by a rheumatologist) in Nova Scotia; Se: 88.4%, PPV: 57.6%. | 710.3 (dermatomyositis), 710.4 (polymyositis)^29^ | M33 (dermatopolymyositis); adapted^30^ | 14 | P: 71.4;  H&P: n<5;  H: n<5. | <5 | 8 |
| **Systemic lupus erythematosus**  **≥1 hospital discharge diagnosis or ≥3 diagnostic billing codes on separate days, any position**  Adapted^31^: ≥1 hospital discharge diagnosis in Nova Scotia; Se: 41.0%, PPV: 99.4%; ≥3 diagnostic billing codes over any time period in Nova Scotia; Se: 79.6%, PPV: 85.1%; codes used: 710.0, M32, M32.1, M32.8, M32.9. | 710.0 (systemic lupus erythematosus)^31^ | M32 (systemic lupus erythematosus), L93 (lupus erythematosus); adapted^30^ | 164 | P: 59.8;  H&P: 24.4;  H: 15.9. | 63 | 94 |
| **Primary biliary cirrhosis**  **≥1 hospital discharge diagnosis or ≥2 diagnostic billing codes on separate days, any position**  Adapted^32^: ≥1 hospital discharge diagnosis in Alberta; Se: 5.9%, PPV: 51%; ≥2 diagnostic billing codes over any time period in Alberta; Se: 88%, PPV: 75%; codes used: 571.6, K74.3. | 571.6 (biliary cirrhosis)^32^ | K74.3 (primary biliary cirrhosis)^32^ | 16 | H: 50.0;  P: n<5;  H&P: n<5. | 5 | 10 |
| **Rheumatoid arthritis**  **≥1 hospital discharge diagnosis or ≥3 diagnostic billing codes on separate days, any position**  Adapted^33^: ≥1 hospital discharge diagnosis in Nova Scotia; Se: 20.7%, PPV: 77.1%; ≥3 diagnostic billing codes over any time period in Nova Scotia; Se: 83.2%, PPV: 53.0%; codes used: 714.0, 714.1, 714.2, M05-M05.9, M06.0, M06.8, M06.9. | 714 (rheumatoid arthritis and other inflammatory polyarthropathies); adapted^33^ | M05 (seropositive rheumatoid arthritis), M06 (other rheumatoid arthritis), M08 (juvenile arthritis); adapted^33^ | 787 | P: 90.5;  H&P: 6.0;  H: 3.6. | 422 | 344 |
| **Sjogren disease**  **≥1 hospital discharge diagnosis or ≥2 diagnostic billing codes on separate days, any position**  Adapted^29^: ≥1 hospital discharge diagnosis (710.2) or ≥2 diagnostic billing codes ≥8 weeks apart in 2 years (or ≥1 if made by a rheumatologist) in Nova Scotia; Se: 95.5%, PPV: 73.0%. | 710.2 (Sicca syndrome)^29^ | M35.0 (Sicca syndrome [Sjögren]); adapted^30^ | 54 | P: 79.6;  H&P: n<5;  H: n<5. | 19 | 33 |
| **Systemic sclerosis**  **≥1 hospital discharge diagnosis or ≥2 diagnostic billing codes on separate days, any position**  Adapted^29^: ≥1 hospital discharge diagnosis (710.1) or ≥2 diagnostic billing codes ≥8 weeks apart in 2 years (or ≥1 if made by a rheumatologist) in Nova Scotia; Se: 80.5%, PPV: 63.5%. | 710.1 (systemic sclerosis)^29^ | M34 (systemic sclerosis); adapted^30^ | 53 | P: 54.7;  H: n<5;  H&P: n<5. | 11 | 41 |
| **Negative control** | | | | | | |
| **Haemorrhoids (negative control)**  **≥1 hospital discharge diagnosis or ≥1 diagnostic billing code, any position**  Adapted^34^: ≥1 hospital discharge diagnosis (455) in the US; Se: 91%, PPV: 97%. | 455 (haemorrhoids)^34^ | I84 (haemorrhoids), K64 (haemorrhoids and perianal venous thrombosis); adapted^34^ | 3,649 | P: 61.8;  H&P: 19.3;  H: 19.0. | 1,007 | 1,193 |

^a^Conditions with counts <100 are grouped into a single “Other” secondary outcome to preserve power: Addison’s disease, dermatomyositis/polymyositis, primary biliary cirrhosis, Sjogren disease and systemic sclerosis. Sources of diagnosis are classified as hospital episodes only (H), physician visits only (P) or both (H&P), and the denominator represents the total count of events. Small non-zero cell sizes (<5 individuals) are masked as per data privacy agreements. Abbreviations: ICD, International Classification of Diseases; PPV, positive predictive value; Se, sensitivity; TIA, transient ischaemic attack.

## Supplementary Table 6. Case definitions and counts for covariates in the cohort.

| **Condition/definition** | **ICD codes** | | **Total count from sources** | **Sources (%)** |
| --- | --- | --- | --- | --- |
|  | **v9** | **v10** |  |  |
| **Chronic kidney disease**  **≥1 hospital discharge diagnosis or ≥2 diagnostic billing codes on separate days within 2 years, any position**  Adapted^35^: ≥1 hospital discharge diagnosis or ≥2 diagnostic billing code within 2 years in Quebec; Se: 92.2%, PPV: 86.1%; codes used: 585, 403, 404, N18, I12, I13. | 585 (chronic renal failure), 403 (hypertensive renal disease), 404 (hypertensive heart and renal disease)^35^ | N18 (chronic kidney disease), I12 (hypertensive renal disease), I13 (hypertensive heart and renal disease)^35^ | 811 | P: 61.9;  H&P: 22.3;  H: 15.8. |
| **Depression**  **≥1 hospital discharge diagnosis or ≥2 diagnostic billing codes on separate days within 1 year, any position**  Adapted^36^: ≥1 hospital discharge diagnosis or ≥2 diagnostic billing codes within 1 year in British Columbia and Alberta; Se: 61.4%, PPV: 69.7%; codes used: 296.2, 296.3, 296.5, 300.4, 309, 311, F20.4, F31.3-F31.5, F32, F33, F34.1, F41.2, F43.2. | 296.2 (major depressive affective disorder, single episode), 296.3 (major depressive affective disorder, recurrent episode), 296.5 (bipolar I disorder, most recent episode [or current] depressed), 300.4 (dysthymic disorder), 309 (adjustment reaction), 311 (depressive disorder, not elsewhere classified)^36^ | F31.3-F31.5 (bipolar affective disorder, current episode depression), F32 (depressive episode), F33 (recurrent depressive disorder), F34 (persistent mood [affective] disorders), F41.2 (mixed anxiety and depressive disorder), F43.2 (adjustment disorders); adapted^36^ | 9,477 | P: 80.7;  H&P: 16.6;  H: 2.7. |
| **Diabetes mellitus**  **≥1 hospital discharge diagnosis or ≥2 diagnostic billing codes on separate days within 1 year, any position**  Adapted^37^: ≥1 hospital discharge diagnosis or ≥2 diagnostic billing code within 1 year in Ontario; Se: 88.4%, PPV: 83.0%; codes used: 250, E10, E11, E13, E14. | 250 (diabetes mellitus)^37^ | E10 (insulin-dependent diabetes mellitus), E11 (non-insulin-dependent diabetes mellitus), E13 (other specified diabetes mellitus), E14 (unspecified diabetes mellitus)^37^ | 2,493 | H&P: 47.3;  P: 44.4;  H: 8.3. |
| **Epilepsy**  **≥1 hospital discharge diagnosis or ≥3 diagnostic billing codes on separate days within 2 years, any position**  Adapted^38^: ≥1 hospital discharge diagnosis or ≥3 diagnostic billing codes within 2 years in Ontario; Se: 76.8%, PPV: 68.2%; codes used: 345, G40. | 345 (epilepsy)^38^ | G40 (epilepsy), G41 (status epilepticus); adapted^38^ | 902 | P: 54.9;  H&P: 32.0;  H: 13.1. |
| **Hyperlipidaemia**  **≥1 hospital discharge diagnosis or ≥2 diagnostic billing codes on separate days within 5 years, any position**  Adapted^39^: ≥1 hospital discharge diagnosis or ≥2 diagnostic billing codes within 5 years in Manitoba (in people with MS); Se: 64.4%, PPV: 65.5%; codes used: 272, E780, E782, E784, E785. | 272 (disorders of lipoid metabolism)^39^ | E78 (disorders of lipoprotein metabolism and other lipidaemias); adapted^39^ | 2,953 | P: 93.8;  H: 3.2;  H&P: 3.0. |
| **Hypertension**  **≥1 hospital discharge diagnosis or ≥2 diagnostic billing codes on separate days within 2 years, any position**  Adapted^39^: ≥1 hospital discharge diagnosis or ≥2 diagnostic billing codes within 2 years in Manitoba (in people with MS); Se: 72.0%, PPV: 81.2%; codes used: 401-405, I10-I13, I15. | 401 (essential hypertension), 402 (hypertensive heart disease), 403 (hypertensive renal disease), 404 (hypertensive heart and renal disease), 405 (secondary hypertension)^39^ | I10 (essential hypertension), I11 (hypertensive heart disease), I12 (hypertensive renal disease), I13 (hypertensive heart and renal disease), I15 (secondary hypertension)^39^ | 6,109 | P: 63.0;  H&P: 30.7;  H: 6.3. |
| **Obesity**  **≥1 hospital discharge diagnosis or ≥2 diagnostic billing codes on separate days, any position**  Adapted^40^: ≥1 hospital discharge diagnosis in Alberta; Se: 7.8%, PPV: 65.9%; codes used: E65-E68. | 278 (overweight, obesity and other hyperalimentation)^41^ | E65 (localized adiposity), E66 (obesity), E67 (other hyperalimentation), E68 (sequelae of hyperalimentation)^40^ | 1,318 | P: 67.2;  H: 21.0;  H&P: 11.8. |
| **Sleep apnoea**  **≥1 hospital discharge diagnosis or diagnostic billing codes on separate days, any position**  Adapted^42^: ≥1 hospital discharge diagnosis or diagnostic billing code in Ontario; Se: 2-58%, Sp (PPV unavailable): 38-99%. | 327.2 (organic sleep apnea)^42^ | G47.3 (sleep apnoea)^42^ | 315 | H: 50.2;  P: 44.8;  H&P: 5.1. |
| **Other covariates** | | | | |
| **Socioeconomic status**: defined from each person’s postal code linked to census derived neighborhood-level income (quintiles)^43^. 98 (0.5%) participants with missing values were imputed by the median. | | | | |
| **Charlson Comorbidity Index** : defined from ≥1 hospital or ≥2 physician visit codes (any position) on separate days within 1 year after removing weights for hemiplegia/paraplegia, which may represent MS manifestations^44^ | | | | |

Sources of diagnosis are classified as hospital episodes only (H), physician visits only (P) or both (H&P), and the denominator represents the total count of events. Abbreviations: ICD, International Classification of Diseases; PPV, positive predictive value; Se, sensitivity; Sp, specificity.

## Supplementary Table 7. Description of people with multiple sclerosis in the study cohort (continued).

| Cohort characteristics (at index date unless specified) | Never exposed to interferon-β (n=16,222) | Ever exposed to interferon-β (n=3,138) |
| --- | --- | --- |
| **Demographics** | | |
| Socioeconomic status, n (%) |  |  |
| Q1 (lowest income quintile) | 3,159 (19.5) | 604 (19.2) |
| Q2 | 3,134 (19.3) | 561 (17.9) |
| Q3 | 3,354 (20.7) | 675 (21.5) |
| Q4 | 3,448 (21.3) | 646 (20.6) |
| Q5 (highest income quintile) | 3,127 (19.3) | 652 (20.8) |
| Calendar year, n (%) | | |
| 1996–1999 | 7,208 (44.4) | 1,325 (42.2) |
| 2000–2005 | 2,976 (18.3) | 929 (29.6) |
| 2006–2011 | 3,055 (18.8) | 667 (21.3) |
| 2012–2017 | 2,983 (18.4) | 217 (6.9) |
| **Contribution to observation time (from index to study end date)** | | |
| Person-years in cohort | 181,536 | 44,388 |
| Years of follow-up per participant, median (Q1-Q3) | 10.3 (4.5-18.2) | 14.7 (9.1-20.2) |
| Deceased, n (%) | 2,978 (18.4) | 232 (7.4) |

## Supplementary Table 8. Hazard ratios for clinical outcomes with unadjusted and adjusted models.

| **Outcome** | **Unadjusted (without binary exposure variable)** | | **Unadjusted (with binary exposure variable)** | | | **Adjusted model 1** | | **Adjusted model 2** | | **Adjusted model 3** | |
| --- | --- | --- | --- | --- | --- | --- | --- | --- | --- | --- | --- |
|  | **HR (95% CI)** | **p-value** | **HR (95% CI)** | **p-value** | | **HR (95% CI)** | **p-value** | **HR (95% CI)** | **p-value** | **HR (95% CI)** | **p-value** |
| **Cardiovascular disease due to atherosclerosis** | | | | | | | | | | | |
| Any | **0.82 (0.74, 0.91)** | **<0.001** | **1.25 (1.08, 1.45)** | **0.003** | | **1.16 (1.00, 1.35)** | **0.043** | **1.18 (1.01, 1.36)** | **0.031** | **1.18 (1.02, 1.37)** | **0.026** |
| Ischaemic heart disease | **0.83 (0.73, 0.94)** | **0.004** | **1.21 (1.02, 1.43)** | **0.033** | | 1.12 (0.94, 1.33) | 0.204 | 1.14 (0.96, 1.36) | 0.144 | 1.15 (0.96, 1.36) | 0.125 |
| Ischaemic stroke | **0.62 (0.50, 0.77)** | **<0.001** | 1.23 (0.93, 1.64) | 0.148 | | 1.16 (0.88, 1.54) | 0.294 | 1.13 (0.85, 1.50) | 0.416 | 1.13 (0.85, 1.50) | 0.414 |
| Peripheral artery disease | 0.86 (0.66, 1.12) | 0.271 | 1.27 (0.87, 1.84) | 0.211 | | 1.16 (0.81, 1.68) | 0.419 | 1.23 (0.84, 1.78) | 0.284 | 1.23 (0.85, 1.79) | 0.275 |
| Congestive heart failure | **0.59 (0.48, 0.72)** | **<0.001** | **1.36 (1.04, 1.78)** | **0.026** | | 1.21 (0.92, 1.58) | 0.164 | 1.27 (0.97, 1.66) | 0.086 | 1.27 (0.97, 1.67) | 0.081 |
| Any stroke | **0.63 (0.51, 0.77)** | **<0.001** | 1.13 (0.86, 1.48) | 0.381 | | 1.06 (0.82, 1.39) | 0.645 | 1.03 (0.79, 1.35) | 0.819 | 1.03 (0.79, 1.35) | 0.806 |
| Atrial fibrillation | **0.62 (0.50, 0.76)** | **<0.001** | 0.91 (0.69, 1.19) | 0.479 | | 0.84 (0.64, 1.10) | 0.207 | 0.82 (0.62, 1.08) | 0.155 | 0.82 (0.62, 1.08) | 0.160 |
| Intracerebral haemorrhage | 0.70 (0.37, 1.32) | 0.269 | 1.25 (0.54, 2.90) | 0.597 | | 1.19 (0.52, 2.73) | 0.685 | 1.13 (0.49, 2.60) | 0.782 | 1.14 (0.49, 2.61) | 0.765 |
| Subarachnoid haemorrhage | 0.74 (0.43, 1.27) | 0.271 | 0.80 (0.38, 1.69) | 0.559 | | 0.79 (0.38, 1.68) | 0.547 | 0.76 (0.36, 1.62) | 0.484 | 0.77 (0.36, 1.63) | 0.488 |
| **Autoimmune disease linked to increased type I interferons** | | | | | | | | | | | |
| Any | **0.64 (0.48, 0.87)** | **0.004** | 0.74 (0.49, 1.10) | | 0.137 | 0.73 (0.49, 1.10) | 0.129 | 0.74 (0.49, 1.11) | 0.139 |  |  |
| Coeliac disease | 0.87 (0.51, 1.46) | 0.588 | 1.07 (0.51, 2.25) | | 0.854 | 1.11 (0.53, 2.35) | 0.785 | 1.17 (0.55, 2.47) | 0.690 |  |  |
| SLE | 0.52 (0.18, 1.48) | 0.218 | 0.97 (0.28, 3.34) | | 0.957 | 1.01 (0.30, 3.45) | 0.986 | 1.05 (0.30, 3.61) | 0.942 |  |  |
| Rheumatoid arthritis | **0.48 (0.30, 0.78)** | **0.003** | 0.72 (0.39, 1.32) | | 0.283 | 0.69 (0.38, 1.27) | 0.233 | 0.67 (0.36, 1.23) | 0.194 |  |  |
| Others | 0.54 (0.25, 1.16) | 0.113 | 0.38 (0.13, 1.16) | | 0.089 | 0.39 (0.13, 1.17) | 0.091 | 0.40 (0.13, 1.22) | 0.108 |  |  |

Estimates represent HRs per 5-year increment in interferon-β exposure duration. All adjusted models are stratified by categorical calendar year at index date (four levels). Model 1 is adjusted for age, sex, socioeconomic status and season. Model 2 is adjusted for covariates in model 1 plus time-updated ever-exposure variables for six disease-modifying therapies. Model 3 is adjusted for covariates in model 2 plus cardiovascular risk factors. Bold characters indicate p<0.05. Abbreviations: CI, confidence interval; HR, hazard ratio; SLE, systemic lupus erythematosus.

## Supplementary Table 9. Sensitivity analyses for the competing risk of death in the primary cardiovascular model (fully adjusted model).

| **Analysis** | **Non-CV deaths treated as failures, n (%)** | **All deaths (non-CV and CV) treated as failures, n (%)** | **Events, n** | **HR (95% CI)** | **p-value** |
| --- | --- | --- | --- | --- | --- |
| Primary analysis | 0 (0.0) | 0 (0.0) | 2,564 | 1.18 (1.02, 1.37) | 0.026 |
| All CV deaths as failures | 0 (0.0) | 119 (6.2) | 2,683 | 1.18 (1.02, 1.36) | 0.028 |
| plus 5% of non-CV deaths as failures | 90 (5.0) | 209 (10.8) | 2,773 | 1.18 (1.02, 1.36) | 0.027 |
| plus 10% of non-CV deaths as failures | 181 (10.0) | 300 (15.5) | 2,864 | 1.13 (0.98, 1.30) | 0.103 |
| plus 15% of non-CV deaths as failures | 271 (15.0) | 390 (20.2) | 2,954 | 1.18 (1.02, 1.35) | 0.023 |

The highest hypothetical cardiovascular failure rate (15%) was greater than the highest cardiovascular disease rate observed across strata of interferon-β treatment duration, i.e. >0 to <0.5: 10.9%, 0.5 to <6.5: 13.0%, 6.5 to <12.5: 12.3%, 12.5 to 22: 7.4%. All analyses are fully adjusted. Cardiovascular disease refers to the composite endpoint of ischaemic heart disease, ischaemic stroke and peripheral artery disease. Abbreviations: CI, confidence interval; CV, cardiovascular; HR, hazard ratio.

## Supplementary Table 10. Warnings and precautions with recommended actions for interferon-β formulations used in the cohort.

| **Drug name** | **Warnings and precautions with recommended actions** | |
| --- | --- | --- |
|  | **US Food and Drug Administration** | **European Medicines Agency** |
| **Cardiovascular adverse events** | | |
| Betaseron^®^/  Betaferon^®^ | **Thrombotic microangiopathy**: “Discontinue BETASERON if clinical symptoms and laboratory findings consistent with thrombotic microangiopathy occur and a relationship to BETASERON is suspected”  **Congestive heart failure**: “Monitor patients with congestive heart failure for worsening of cardiac symptoms; consider discontinuation of BETASERON if worsening of congestive heart failure occurs” | **Thrombotic microangiopathy**: “If […] suspected, prompt treatment is required (in case of thrombotic microangiopathy considering plasma exchange) and immediate discontinuation of Betaferon is recommended”  **Pre-existing cardiac disease**: “Patients with pre-existing significant cardiac disease, such as congestive heart failure, coronary artery disease or arrhythmia, should be monitored for worsening of their cardiac condition, particularly during initiation of treatment with Betaferon” |
| Extavia^®^ | **Thrombotic microangiopathy**: “Discontinue EXTAVIA if clinical symptoms and laboratory findings consistent with thrombotic microangiopathy occur”  **Congestive heart failure**: “Monitor patients with congestive heart failure for worsening of cardiac symptoms; consider discontinuation of EXTAVIA if worsening of congestive heart failure occurs” | **Thrombotic microangiopathy**: “If […] diagnosed, and a relationship to Extavia is suspected, prompt treatment is required (in case of thrombotic microangiopathy considering plasma exchange) and immediate discontinuation of Extavia is recommended”  **Pre-existing cardiac disease**: “Patients with pre-existing significant cardiac disease, such as congestive heart failure, coronary artery disease or arrhythmia, should be monitored for worsening of their cardiac condition, particularly during initiation of treatment with Extavia” |
| Rebif^®^ | **Thrombotic microangiopathy**: “Discontinue REBIF if clinical symptoms and laboratory findings consistent with thrombotic microangiopathy occur, and manage as clinically indicated” | **Thrombotic microangiopathy**: “If thrombotic microangiopathy is diagnosed, prompt treatment is required (considering plasma exchange) and immediate discontinuation of Rebif is recommended”  **Pre-existing cardiac disease**: “Patients with cardiac disease, such as angina, congestive heart failure or arrhythmia, should be closely monitored for worsening of their clinical condition during initiation of therapy with interferon beta-1a” |
| Avonex^®^ | **Thrombotic microangiopathy**: “Discontinue AVONEX if clinical symptoms and laboratory findings  consistent with thrombotic microangiopathy occur”  **Congestive heart failure**: “Monitor patients with pre-existing significant cardiac disease for worsening of cardiac symptoms” | **Thrombotic microangiopathy**: “If thrombotic microangiopathy is diagnosed, prompt treatment is required (considering plasma exchange) and immediate discontinuation of AVONEX is recommended”  **Pre-existing cardiac disease**: “Patients with cardiac disease, such as angina, congestive heart failure or arrhythmia, should be closely monitored for worsening of their clinical condition during treatment with AVONEX” |
| Plegridy^®^ | **Thrombotic microangiopathy**: “Discontinue PLEGRIDY if clinical symptoms and laboratory findings consistent with thrombotic microangiopathy occur”  **Congestive heart failure**: “Monitor patients with pre-existing significant cardiac disease for worsening of cardiac symptoms” | **Thrombotic microangiopathy**: “If thrombotic microangiopathy is diagnosed, prompt treatment is required (considering plasma exchange) and immediate discontinuation of peginterferon beta-1a is recommended”  **Pre-existing cardiac disease**: “Patients with pre-existing significant cardiac disease, such as congestive heart failure, coronary artery disease or arrhythmia should be monitored for worsening of their cardiac condition, particularly during initiation of treatment” |
| **Autoimmune adverse events** | | |
| Betaseron^®^/  Betaferon^®^ | **Drug-induced lupus erythematosus**: “Discontinue BETASERON if patients develop new characteristic signs and symptoms.”  **Autoimmune hepatitis**: “Monitor liver function tests and signs and symptoms of hepatic injury; consider discontinuing BETASERON if serious hepatic injury occurs” | None |
| Extavia^®^ | **Drug-induced lupus erythematosus**: “Discontinue EXTAVIA if patients develop new characteristic signs and symptoms”  **Autoimmune hepatitis**: “Monitor patients for signs and symptoms of hepatic injury. Consider discontinuing EXTAVIA if serum transaminase levels significantly increase, or if they are associated with clinical symptoms such as jaundice” | None |
| Rebif^®^ | None | None |
| Avonex^®^ | **Autoimmune disorders**: “Consider discontinuation of AVONEX if new autoimmune disorder occurs” | **Autoimmune hepatitis**: “Patients should be monitored for signs of hepatic injury and caution exercised when interferons are used concomitantly with other medicinal products associated with hepatic injury” |
| Plegridy^®^ | **Autoimmune disorders**: “Consider discontinuation of PLEGRIDY if a new autoimmune disorder occurs” | **Autoimmune hepatitis**: “Patients should be monitored for signs of hepatic injury” |

Product information can be retrieved from <https://www.accessdata.fda.gov/scripts/cder/daf/index.cfm> (US Food and Drug Administration) and <https://www.ema.europa.eu/en/medicines> (European Medicines Agency).

# SUPPLEMENTARY FIGURES

## Supplementary Fig. 1. Graphical summary of the study follow-up.


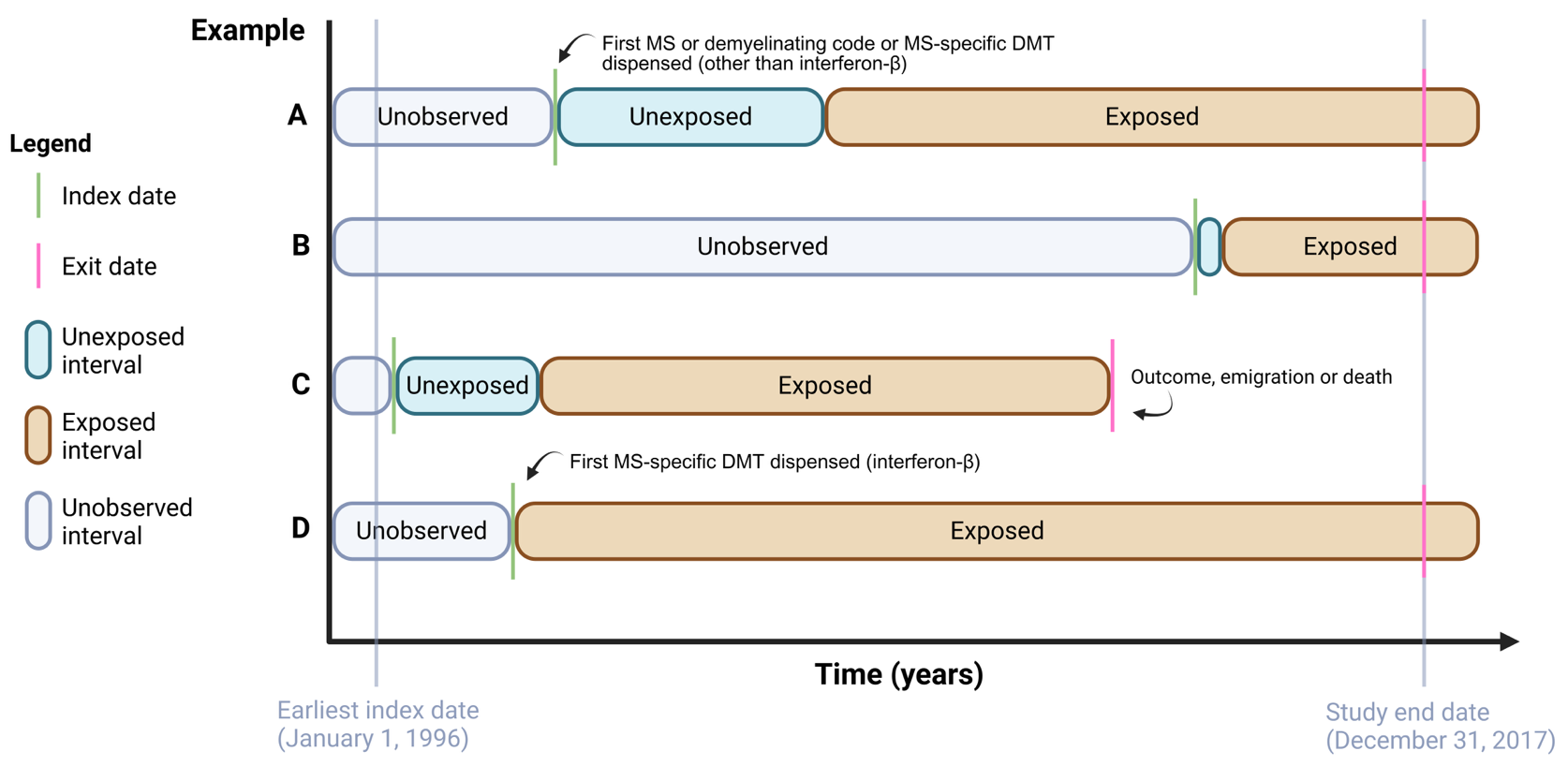


Graphical summary of the study follow-up using four hypothetical examples (A-D). In A, the participant is included at the time of first MS or demyelinating code or MS-specific DMT dispensed (index date), but no interferon-β therapy is dispensed at this time. The participant is considered unobserved prior to the index date, then unexposed from the index date until an interferon-β medication is later dispensed. In B, the participant is included following similar patterns as A, but the inclusion occurs later during the observation time window and the unexposed time is shorter. In C, the participant has a shorter unobserved time before inclusion (i.e., before the index date) and follow-up ends before the study end date due to the occurrence of an outcome of interest, emigration or death. In D, the participant is included at the time an interferon-β medication is dispensed and is considered exposed from the time of inclusion onwards. ‘Unobserved’ time periods contribute towards prevalent conditions but not incident outcomes. Abbreviations: DMT, disease-modifying therapy; MS, multiple sclerosis. Created in BioRender. Rioux, B. (2025): https://BioRender.com/mqcr35e.

## Supplementary Fig. 2. Age-standardised cardiovascular disease rates by treatment group.


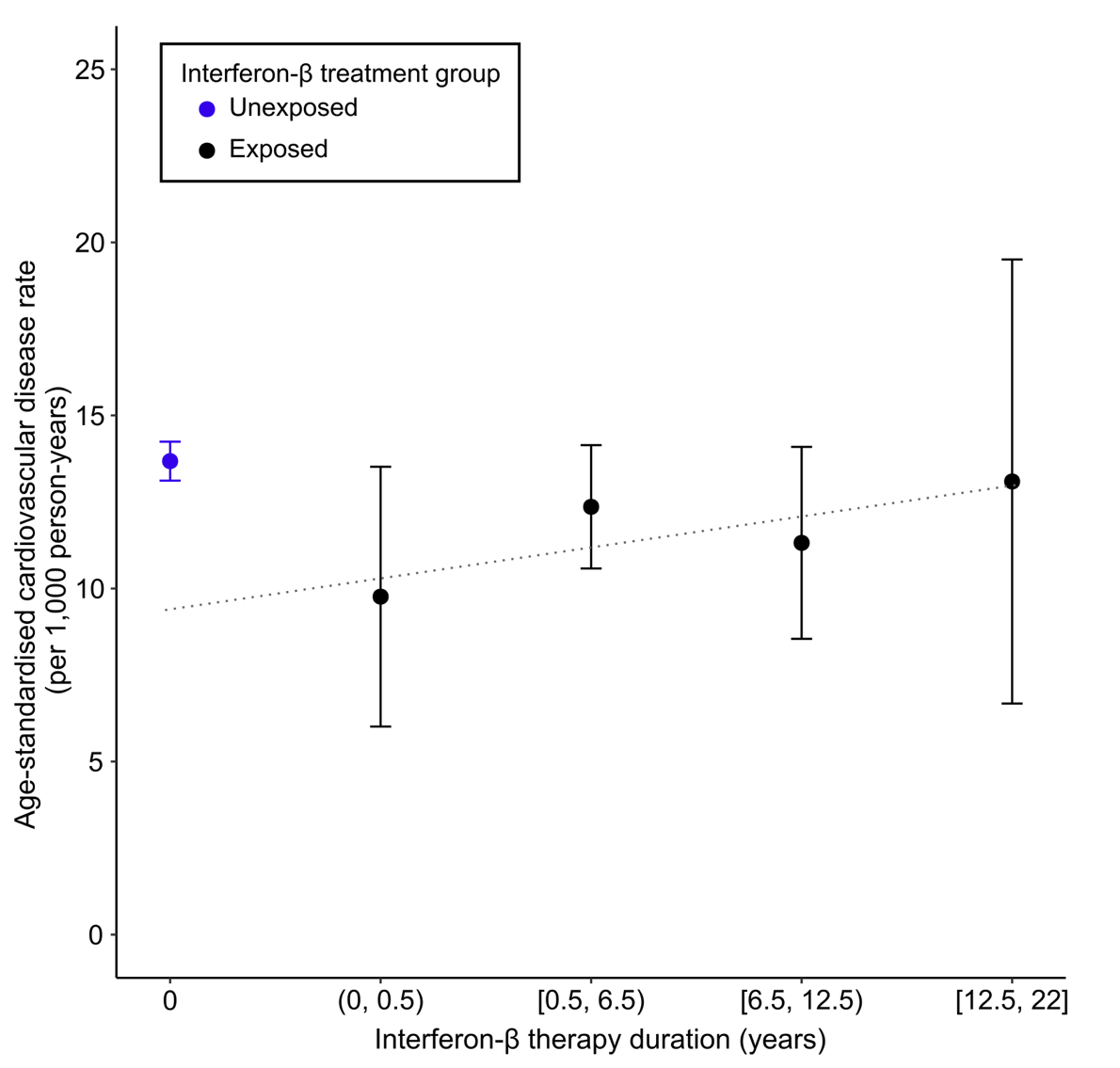


Age-standardised cardiovascular disease rates (primary cardiovascular disease outcome) per 1,000 person-years in unexposed (blue) and exposed (black) participants across strata of interferon-β therapy duration (years). Direct standardisation was applied per age year (time-updated) using the full cohort as reference group. The observed event rate is higher in the untreated group because of allocation bias (on average, untreated participants are older and have a greater prevalence of cardiovascular risk factors). The dotted line represents the fitted regression line across exposure groups (coded 1-4), extrapolated back to zero. The y-axis intercept represents the expected rate at the point of starting the exposure in those exposed after evacuating allocation bias. The rate of cardiovascular disease is increased by 3.6 per 1,000 person-years of follow-up in the highest exposure strata (i.e., 12.5 to 22 years) as compared to the projected rate if unexposed (13.0 vs 9.4 per 1,000 person-years). For intervals, closed and open brackets respectively indicate the endpoint is included and excluded.

## Supplementary Fig. 3. Forest plots of adjusted hazard ratios for cardiovascular and autoimmune diseases after (A) starting follow-up at index date plus 30 days, (B) removing participants with an index date shifted forward, (C) removing strokes occurring within the first 30 days of follow-up, and (D) removing strokes only defined through physician visits.


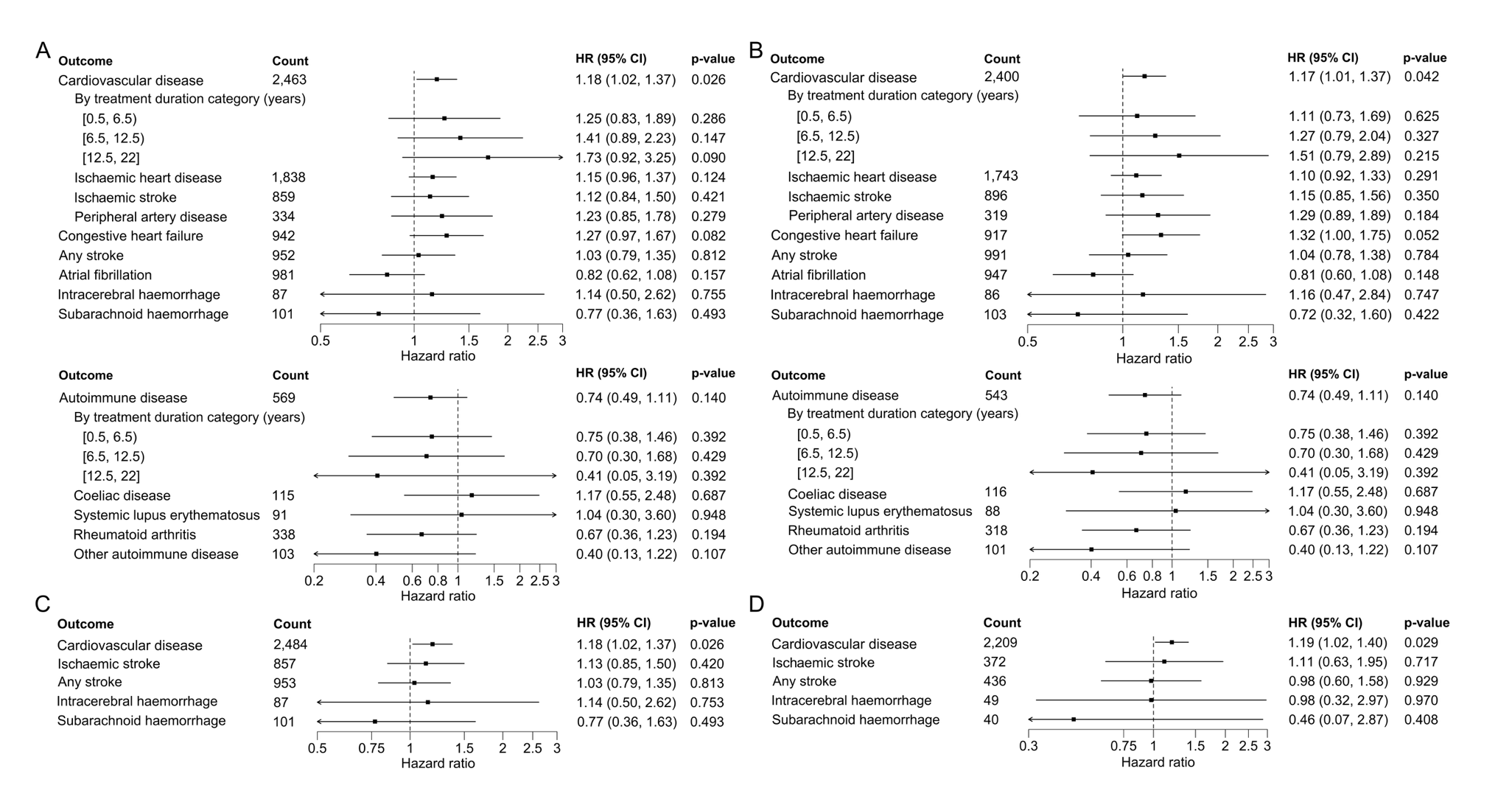


Estimates represent HRs per 5-year increment in interferon-β therapy duration from fully adjusted Cox regression models in the cohort (N=19,360 participants in the full cohort). For primary outcomes, categorical estimates represent HRs for levels of interferon-β therapy duration (in years) using [0, 0.5) as the reference. Axes are on the log10 scale. For intervals, closed and open brackets respectively indicate the endpoint is included and excluded. Abbreviations: CI, confidence interval; HR, hazard ratio.

# SUPPLEMENTARY REFERENCES

1. Edvardsen K, Bjånesøy T, Hellesen A*, et al*. Peripheral Blood Cells from Patients with Autoimmune Addison's Disease Poorly Respond to Interferons In Vitro, Despite Elevated Serum Levels of Interferon-Inducible Chemokines. *J Interferon Cytokine Res*. Oct 2015;35(10):759-70. doi:10.1089/jir.2014.0171

2. Chan WB, Chow CC, Cockram CS. Interferon alpha treatment and endocrine disease. *J R Soc Med*. Oct 2003;96(10):481-5. doi:10.1177/014107680309601003

3. Sallese M, Efthymakis K, Marchioni M*, et al*. Gene Expression Profiling in Coeliac Disease Confirmed the Key Role of the Immune System and Revealed a Molecular Overlap with Non-Celiac Gluten Sensitivity. *Int J Mol Sci*. Apr 24 2023;24(9)doi:10.3390/ijms24097769

4. Cammarota G, Cuoco L, Cianci R, Pandolfi F, Gasbarrini G. Onset of coeliac disease during treatment with interferon for chronic hepatitis C. *Lancet*. Oct 28 2000;356(9240):1494-5. doi:10.1016/s0140-6736(00)02880-4

5. Walsh RJ, Kong SW, Yao Y*, et al*. Type I interferon-inducible gene expression in blood is present and reflects disease activity in dermatomyositis and polymyositis. *Arthritis Rheum*. Nov 2007;56(11):3784-92. doi:10.1002/art.22928

6. Greenberg SA, Higgs BW, Morehouse C*, et al*. Relationship between disease activity and type 1 interferon- and other cytokine-inducible gene expression in blood in dermatomyositis and polymyositis. *Genes & Immunity*. 2012/04/01 2012;13(3):207-213. doi:10.1038/gene.2011.61

7. Arshanapalli A, Shah M, Veerula V, Somani A-K. The role of type I interferons and other cytokines in dermatomyositis. *Cytokine*. 2015/06/01/ 2015;73(2):319-325. doi:10.1016/j.cyto.2014.11.026

8. Shiba H, Takeuchi T, Isoda K*, et al*. Dermatomyositis as a complication of interferon-α therapy: a case report and review of the literature. *Rheumatology International*. 2014/09/01 2014;34(9):1319-1322. doi:10.1007/s00296-014-2984-4

9. Postal M, Vivaldo JF, Fernandez-Ruiz R, Paredes JL, Appenzeller S, Niewold TB. Type I interferon in the pathogenesis of systemic lupus erythematosus. *Curr Opin Immunol*. Dec 2020;67:87-94. doi:10.1016/j.coi.2020.10.014

10. Ho V, McLean A, Terry S. Severe Systemic Lupus Erythematosus Induced by Antiviral Treatment for Hepatitis C. *JCR: Journal of Clinical Rheumatology*. 2008;14(3)

11. D'Amico E, Paroli M, Fratelli V*, et al*. Primary biliary cirrhosis induced by interferon-alpha therapy for hepatitis C virus infection. *Dig Dis Sci*. Oct 1995;40(10):2113-6. doi:10.1007/bf02208992

12. Takii Y, Nakamura M, Ito M*, et al*. Enhanced expression of type I interferon and toll-like receptor-3 in primary biliary cirrhosis. *Laboratory Investigation*. 2005/07/01 2005;85(7):908-920. doi:10.1038/labinvest.3700285

13. Papadopoulos VE, Skarlis C, Evangelopoulos M-E, Mavragani CP. Type I interferon detection in autoimmune diseases: challenges and clinical applications. *Expert Review of Clinical Immunology*. 2021/08/03 2021;17(8):883-903. doi:10.1080/1744666X.2021.1939686

14. Salzmann RJS, Krötz C, Mocan T*, et al*. Increased type-I interferon level is associated with liver damage and fibrosis in primary sclerosing cholangitis. *Hepatol Commun*. Mar 1 2024;8(3)doi:10.1097/hc9.0000000000000380

15. Lin CMA, Isaacs JD, Cooles FAH. Role of IFN-α in Rheumatoid Arthritis. *Current Rheumatology Reports*. 2024/02/01 2024;26(2):37-52. doi:10.1007/s11926-023-01125-6

16. Passos de Souza E, Evangelista Segundo PT, José FF, Lemaire D, Santiago M. Rheumatoid arthritis induced by alpha-interferon therapy. *Clin Rheumatol*. 2001;20(4):297-9. doi:10.1007/pl00011206

17. Nordmark G, Alm GV, Rönnblom L. Mechanisms of Disease: primary Sjögren's syndrome and the type I interferon system. *Nature Clinical Practice Rheumatology*. 2006/05/01 2006;2(5):262-269. doi:10.1038/ncprheum0173

18. Unoki H, Moriyama A, Tabaru A, Masumoto A, Otsuki M. Development of Sjögren's syndrome during treatment with recombinant human interferon-alpha-2b for chronic hepatitis C. *Journal of Gastroenterology*. 1996/10/01 1996;31(5):723-727. doi:10.1007/BF02347624

19. Kakkar V, Assassi S, Allanore Y*, et al*. Type 1 interferon activation in systemic sclerosis: a biomarker, a target or the culprit. *Curr Opin Rheumatol*. Nov 1 2022;34(6):357-364. doi:10.1097/bor.0000000000000907

20. Solans R, Bosch JA, Esteban I, Vilardell M. Systemic sclerosis developing in association with the use of interferon alpha therapy for chronic viral hepatitis. *Clin Exp Rheumatol*. Sep-Oct 2004;22(5):625-8.

21. Tu K, Nieuwlaat R, Cheng SY*, et al*. Identifying Patients With Atrial Fibrillation in Administrative Data. *Canadian Journal of Cardiology*. 2016/12/01/ 2016;32(12):1561-1565. doi:10.1016/j.cjca.2016.06.006

22. Tu K, Wang M, Young J*, et al*. Validity of Administrative Data for Identifying Patients Who Have Had a Stroke or Transient Ischemic Attack Using EMRALD as a Reference Standard. *Canadian Journal of Cardiology*. 2013/11/01/ 2013;29(11):1388-1394. doi:10.1016/j.cjca.2013.07.676

23. Schultz SE, Rothwell DM, Chen Z, Tu K. Identifying cases of congestive heart failure from administrative data: a validation study using primary care patient records. *Chronic Dis Inj Can*. Jun 2013;33(3):160-6.

24. Tu K, Mitiku T, Lee DS, Guo H, Tu JV. Validation of physician billing and hospitalization data to identify patients with ischemic heart disease using data from the Electronic Medical Record Administrative data Linked Database (EMRALD). *Can J Cardiol*. Aug-Sep 2010;26(7):e225-8. doi:10.1016/s0828-282x(10)70412-8

25. Hong Y, Sebastianski M, Makowsky M, Tsuyuki R, McMurtry MS. Administrative data are not sensitive for the detection of peripheral artery disease in the community. *Vascular Medicine*. 2016;21(4):331-336. doi:10.1177/1358863x16631041

26. Jacob-Brassard J, Al-Omran M, Stukel TA, Mamdani M, Lee DS, De Mestral C. Validation of Diagnosis and Procedure Codes for Revascularization for Peripheral Artery Disease in Ontario Administrative Databases. *Clin Invest Med*. Jun 14 2021;44(2):E36-43. doi:10.25011/cim.v44i2.36354

27. Sekhon SS, Crick K, Myroniuk TW*, et al*. Adrenal Insufficiency: Investigating Prevalence and Healthcare Utilization Using Administrative Data. *J Endocr Soc*. Apr 1 2022;6(4):bvab184. doi:10.1210/jendso/bvab184

28. Duerksen DR, Lix LM, Leslie WD. Development of an administrative definition for celiac disease. *BMC Res Notes*. Oct 17 2019;12(1):661. doi:10.1186/s13104-019-4693-2

29. Bernatsky S, Linehan T, Hanly JG. The accuracy of administrative data diagnoses of systemic autoimmune rheumatic diseases. *J Rheumatol*. Aug 2011;38(8):1612-6. doi:10.3899/jrheum.101149

30. Kuan V, Denaxas S, Gonzalez-Izquierdo A*, et al*. A chronological map of 308 physical and mental health conditions from 4 million individuals in the English National Health Service. *The Lancet Digital Health*. 2019;1(2):e63-e77. doi:10.1016/S2589-7500(19)30012-3

31. Hanly JG, Thompson K, Skedgel C. Identification of patients with systemic lupus erythematosus in administrative healthcare databases. *Lupus*. Nov 2014;23(13):1377-82. doi:10.1177/0961203314543917

32. Myers RP, Shaheen AA, Fong A*, et al*. Validation of coding algorithms for the identification of patients with primary biliary cirrhosis using administrative data. *Can J Gastroenterol*. Mar 2010;24(3):175-82. doi:10.1155/2010/237860

33. Hanly JG, Thompson K, Skedgel C. The use of administrative health care databases to identify patients with rheumatoid arthritis. *Open Access Rheumatol*. 2015;7:69-75. doi:10.2147/OARRR.S92630

34. Delco F, Sonnenberg A. Associations between hemorrhoids and other diagnoses. *Dis Colon Rectum*. Dec 1998;41(12):1534-41; discussion 1541-2. doi:10.1007/BF02237302

35. Roy L, Zappitelli M, White-Guay B, Lafrance JP, Dorais M, Perreault S. Agreement Between Administrative Database and Medical Chart Review for the Prediction of Chronic Kidney Disease G category. *Can J Kidney Health Dis*. 2020;7:2054358120959908. doi:10.1177/2054358120959908

36. Doktorchik C, Patten S, Eastwood C*, et al*. Validation of a case definition for depression in administrative data against primary chart data as a reference standard. *BMC Psychiatry*. 2019/01/07 2019;19(1):9. doi:10.1186/s12888-018-1990-6

37. Lipscombe LL, Hwee J, Webster L, Shah BR, Booth GL, Tu K. Identifying diabetes cases from administrative data: a population-based validation study. *BMC Health Services Research*. 2018/05/02 2018;18(1):316. doi:10.1186/s12913-018-3148-0

38. Tu K, Wang M, Jaakkimainen RL*, et al*. Assessing the validity of using administrative data to identify patients with epilepsy. *Epilepsia*. Feb 2014;55(2):335-43. doi:10.1111/epi.12506

39. Marrie RA, Yu BN, Leung S*, et al*. Rising prevalence of vascular comorbidities in multiple sclerosis: validation of administrative definitions for diabetes, hypertension, and hyperlipidemia. *Multiple Sclerosis Journal*. 2012;18(9):1310-1319. doi:10.1177/1352458512437814

40. Martin B-J, Chen G, Graham M, Quan H. Coding of obesity in administrative hospital discharge abstract data: accuracy and impact for future research studies. *BMC Health Services Research*. 2014/02/13 2014;14(1):70. doi:10.1186/1472-6963-14-70

41. Kuhle S, Kirk SFL, Ohinmaa A, Veugelers PJ. Comparison of ICD code-based diagnosis of obesity with measured obesity in children and the implications for health care cost estimates. *BMC Medical Research Methodology*. 2011/12/21 2011;11(1):173. doi:10.1186/1471-2288-11-173

42. McIsaac DI, Gershon A, Wijeysundera D, Bryson GL, Badner N, van Walraven C. Identifying Obstructive Sleep Apnea in Administrative Data: A Study of Diagnostic Accuracy. *Anesthesiology*. 2015;123(2):253-263. doi:10.1097/aln.0000000000000692

43. Ng HS, Zhu F, Kingwell E*, et al*. Characteristics of a population-based multiple sclerosis cohort treated with disease-modifying drugs in a universal healthcare setting. *Expert Rev Neurother*. Jan 2021;21(1):131-140. doi:10.1080/14737175.2021.1847085

44. Needham DM, Scales DC, Laupacis A, Pronovost PJ. A systematic review of the Charlson comorbidity index using Canadian administrative databases: a perspective on risk adjustment in critical care research. *Journal of Critical Care*. 2005/03/01/ 2005;20(1):12-19. doi:10.1016/j.jcrc.2004.09.007
